# The chemical profiling of *Salvia plebeia* during different growth periods and the biosynthesis of its main flavonoids ingredients

**DOI:** 10.3389/fpls.2023.1228356

**Published:** 2023-08-14

**Authors:** Yiqun Dai, Ziyu Ye, Hui Liu, Ruirui Zhu, Lanlan Sun, Shuai Li, Guoyong Xie, Yan Zhu, Yucheng Zhao, Minjian Qin

**Affiliations:** ^1^ Department of Resources Science of Traditional Chinese Medicines, School of Traditional Chinese Pharmacy, China Pharmaceutical University, Nanjing, China; ^2^ School of Pharmacy, Bengbu Medical College, Bengbu, China; ^3^ Yangzhou Center for Food and Drug Control, Yangzhou, China

**Keywords:** *Salvia plebeia*, LC-MS/MS, transcriptome, flavonoid biosynthesis, dynamic distribution, harvest time

## Abstract

*Salvia plebeia* (Lamiaceae) is a valuable medicinal plant widely distributed across Asia and Oceania. However, the composition and accumulation patterns of its active ingredients in different organs during the growth and their biosynthetic mechanism remain unknown. Therefore, we conducted metabolite profiling, transcriptomic analysis, and biological functional verification to explore the distribution, accumulation, and biosynthesis mechanisms of flavonoids in *S. plebeia.* We identified 70 metabolites including 46 flavonoids, 16 phenolic acids, seven terpenoids, and one organic acid, of which 21 were previously unreported in *S. plebeia*. Combining metabolomic-transcriptomic analysis and biological functional verification, we identified the key genes involved in biosynthesis of its main active ingredients, hispidulin and homoplantaginin, including *SpPAL*, *SpC4H*, *Sp4CL2*, *Sp4CL5*, *SpCHS1*, *SpCHI*, *SpFNS*, *SpF6H1*, *SpF6OMT1*, *SpF6OMT2*, *SpUGT1*, *SpUGT2*, and *SpUGT3*. Using the identified genes, we reconstructed the hispidulin and homoplantaginin biosynthesis pathways in *Escherichia coli*, and obtained a yield of 5.33 and 3.86 mg/L for hispidulin and homoplantaginin, respectively. Our findings provide valuable insights into the changes in chemical components in different organs of *S. plebeia* during different growth and harvest stages and establishes a foundation for identifying and synthesizing its active components.

## Introduction

*Salvia plebeia* R. Br. is a valuable medicinal plant belonging to the genus *Salvia* in Labiatae, which widely distributed in China, Japan, Korea, Australia, and India. It has long history used for traditional herb medicine in the *Compendium of Materia Medica* above 490 years ago in the Ming Dynasty of China. Traditional Chinese Medicines (TCMs) believes that *S. plebeia* can clear heat and detoxify the toxins. Consequently, it has been included in the China Pharmacopoeia cure for bronchitis, hemorrhoids, and nephritis (Liang et al., 2020). Modern pharmaceutical findings have further reported that *S. plebeia* extract possesses anticancer, immunomodulatory, anti-inflammatory, antioxidant, and antiviral activities (Choi et al., 2020; Shin et al., 2020; Liang et al., 2021).

TCMs pharmacologists acknowledged that the different pharmacological effects of plant medicines are attributed to their potent secondary metabolites. In addition, the chemical components of TCMs are influenced by various factors, such as genetic background, organ and tissue specificity, growth stages, cultivation methods, harvest times, and processing/storage condition (Zhu et al., 2014). While the information is limited about the compositions and dynamic accumulation patterns of its active ingredients in different organs during the growth and development of *S. plebeia.* According to China Pharmacopoeia, the dried aerial part of *S. plebeia* should be harvested in summer (Commission, C.P., 1997). So that, most of the medicinal materials of the herb in Chinese herbal materials markets are originated from the dried aerial part of the plant possessing the flowers or fruits (Wang et al., 2010). However, literature also reported that in some place of Eastern China, the fresh seedlings of the plant without elongating stem, those are mainly basal leaves collected in spring, were used as folk medicine for treating senile chronic bronchitis and mastitis (Jiang Su Food and Drug Administration, 2016) and had good cure effects. Why do different medicinal parts and harvesting time of the same plant produce different therapeutic effects, and what are the molecular mechanisms involved in, which aroused our research interest.

The phytochemical investigations have showed that *S. plebeia* contains sesquiterpenoids, flavonoids, phenylpropanoids, phenolic acids and diterpenoids, etc (Liang et al., 2020). Previous reports indicated that flavonoids, especially hispidulin and homoplantaginin, are the main bioactive compounds of *S. plebeia*. Recent studies have demonstrated that hispidulin and homoplantaginin have multiple pharmacological effects, such as anticancer, anti-inflammatory, neuroprotective, anti-epileptic, alleviated vascular endothelial cell apoptosis (Lin et al., 2015; He et al., 2016; An et al., 2019; Liu et al., 2020; Fan et al., 2023). However, the difficulties in purification and the challenges in chemical synthesize limit their further development and wider application in clinical studies. Biosynthesis is expected to solve this problem with its advantages of low cost, high yield and environmental friendliness. Whereas, the information on flavonoid biosynthesis in *S. plebeia*, including relevant biosynthetic enzymes, genes and their regulation mechanisms is also unclear. Based on previous reports, the biosynthetic pathway of homoplantaginin can be predicted (Li et al., 2019; Nabavi et al., 2020; Pei et al., 2022). The homoplantaginin biosynthesis pathway is initiated by *PAL*, *PAL* converts phenylalanine to cinnamic acid. Subsequently, cinnamic acid reacts with cinnamon-4-hydroxylase (*C4H*) and 4-coumaryl-CoA ligase (*4CL*) to produce 4-coumaryl-CoA, the precursor compound of naringin chalcone. Chalcone synthase (*CHS*) is the starting enzyme for the synthesis of flavonoids, and its product naringin chalcone is transformed into naringenin by chalcone isomerase (*CHI*). Then apigenin is produced by flavone synthase II (*FNS*). Apigenin is converted to hispidulin under the flavone 6-hydroxylase (*F6H*) and flavone 6-O-methyltransferase (*F6OMT*). Eventually, UDP-glycosyltransferase (*UGT*) converts hispidulin into homoplantaginin. Although the key enzymes for homoplantaginin biosynthesis can be predicted from other species plant biosynthetic pathway, the complete biosynthetic pathway of homoplantaginin has not been reported in plants.

Here, we systematically analyzed the chemical profiles of different organs in *S. plebeia* at different developmental stages using UPLC-Q-TOF-MS/MS and HPLC-DAD methods. In addition, a transcriptome dataset of *S. plebeia* was constructed for the first time to analyze the expression levels of genes related to flavonoid biosynthesis in different organs at different developmental stages. Finally, all candidate genes were functionally verified, and a synthetic biology platform was constructed to obtain main flavonoids of *S. plebeia*. This result provides insights into the chemical profiling of *S. plebeia* and would help in the further exploitation.

## Materials and methods

### Plant materials and chemical reagents

**Figure 1:**
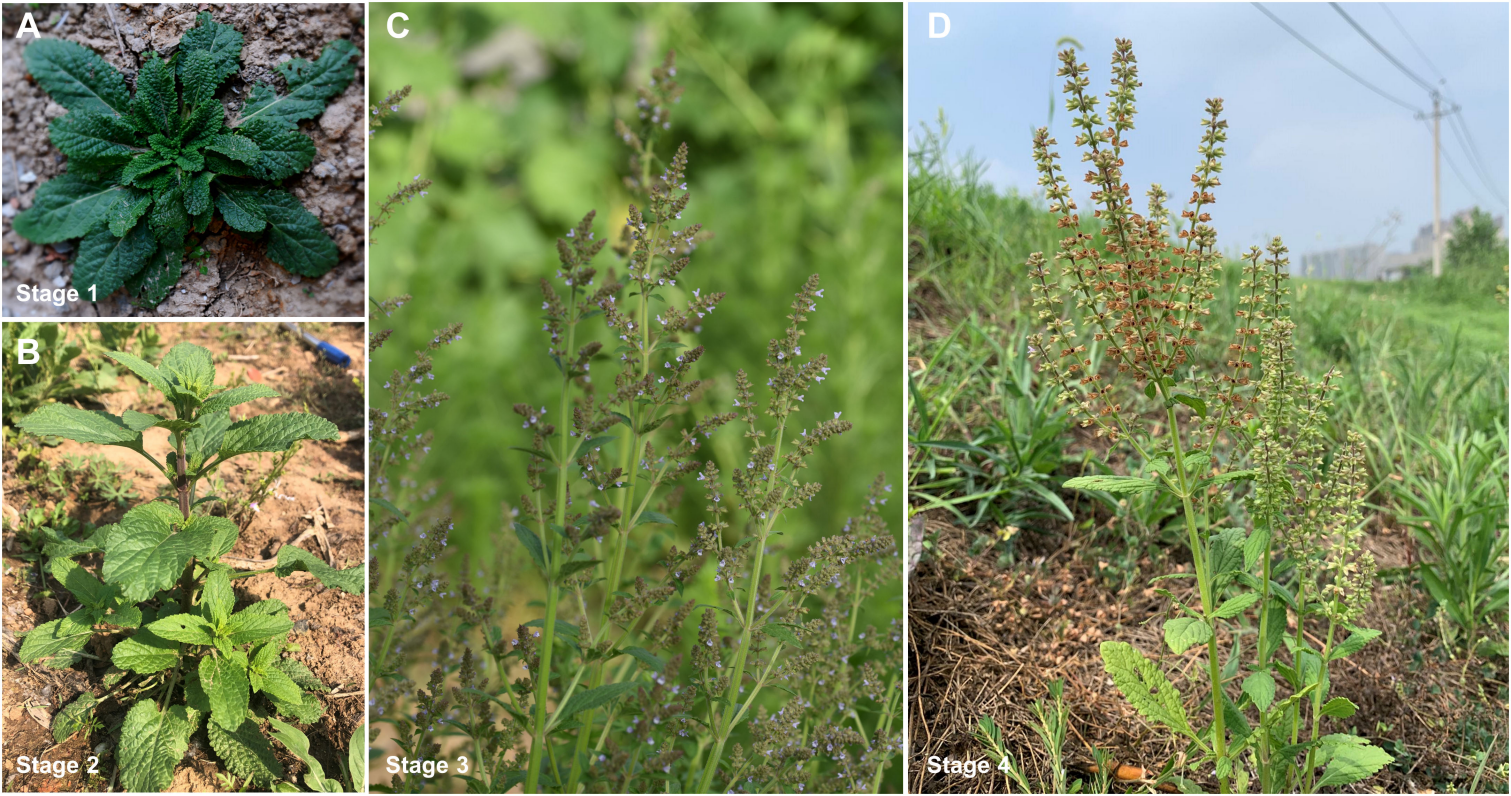
*S. plebeia* collected at different growth stages. **(A)** Samples collected at Mar. 2 (stage 1 (basal leaf stage), harvest parts: leaves (L1), root (R1)). **(B)** Samples collected at Apr. 7 (stage 2 (stem elongation stage), harvest parts: leaves (L2), stem (S2), root (R2)). **(C)** Samples collected at May 1 (stage 3 (flower stage), harvest parts: leaves (L3), stem (S3), flower (F3), root (R3)). **(D)** Samples collected at Jun. 1, (stage 4 (ripening stage), harvest parts: leaves (L4), stem (S4), root (R4)).

The plant materials of *S. plebeia* were obtained from the Medicinal Botanic Garden of China Pharmaceutical University, Nanjing, China (118.83E, 31.95N). The samples were collected at four representative stages—the basal leaf stage (stage 1, harvest parts: leaves (L1) and root (R1)); stem elongation stage (stage 2, harvest parts: leaves (L2), stem (S2), and root (R2)); flower stage (stage 3, harvest parts: leaves (L3), stem (S3), flower (F3), and root (R3)); and ripening stage (stage 4, harvest parts: leaves (L4), stem (S4), and root (R4)) (**Figure 1**). All organs were chopped and snap frozen in liquid nitrogen, then stored at -80°C. Eight biological replicates were sampled for metabolomics assay. Three biological replicas were sampled for transcriptomic assay.

The reference standards, 6-hydroxyluteolin 7-*O*-β-glucoside, nepitrin, luteolin 7-*O*-β-glucoside, homoplantaginin, apigenin 7-*O*-β-glucoside, luteolin, nepetin, 6-methoxynaringenin, apigenin, hispidulin, phenylalanine, cinnamic acid, 4-coumaric acid, tyrosine, naringenin chalcone, naringenin, scutellarein, UDP-glucose, and NADPH had >98% purity and were commercially available (Push, Chengdu, China).

### UPLC-Q-TOF-MS/MS and HPLC-DAD conditions

All samples were crushed, weighed 0.5 g into 10 mL of 70% methanol (0.02 mg/mL baicalin and 0.01 mg/mL irigenin as internal standards for UPLC-Q-TOF-MS/MS assay). The mixed solution was sonicated for 30 min. After ultrasonic extraction, the extraction solution centrifuged at 10,000 rpm for 30 min. Then, the supernatant was filtered by a 0.22µm membrane filter. The solutions were injection into the column for UPLC-Q-TOF-MS/MS and HPLC-DAD analysis.

The UPLC-Q-TOF-MS/MS analysis was using an AB SCIEX TripleToF® 5600 mass analyzer (Redwood City, CA, USA), in negative ion modes. A C_18_ reversed phase column (50 mm × 2.1 mm, 1.5 µm, Thermo Scientific, USA) was used for UPLC analysis with 40°C column temperature. The gradient contains solvents A (0.1% formic acid in water) and solvents B (acetonitrile) with the gradient as follows: 0 min, 12% B; 3 min,16% B; 5 min, 16.5% B; 8 min, 18% B; 9 min, 27% B; 12 min, 33% B; 13 min, 50% B; and 14 min, 95% B. The flow rate was maintained at 0.4 mL/min. MS survey scan of 100-2000 Da; ion source heater, 550°C; ion spray voltage, 4500 V; and collision energy, 44 V. The Peakview Software (version 1.2.0.3, AB SCIEX, Redwood City, CA, USA) were used to analyzed MS/MS data.

The contents of 10 main flavonoids (6-hydroxyluteolin 7-*O*-β-glucoside, nepitrin, luteolin 7-*O*-β-glucoside, apigenin 7-*O*-β-glucoside, homoplantaginin, luteolin, nepetin, 6-methoxynaringenin, apigenin, and hispidulin) in each sample of *S. plebeia* were performed by external standard method, using a 1290 HPLC instrument (Agilent Technologies, Cambridge, USA) with DAD wavelength at 280 nm and 342 nm. The HPLC condition were consistent with those for the UPLC-Q-TOF-MS/MS analysis. Three replicates per sample.

### Illumina sequencing and transcriptomic analysis

Total RNA was isolated from *S. plebeia* using the RNA simple total RNA kit (TIANGEN, Beijing, China). A cDNA library was then constructed and sequenced on an Illumina NovaSeq 6000 platform (Illumina, San Diego, CA, USA). All raw reads were uploaded to NCBI at SRA database (PRJNA952802) and include 21 accession items (SRR24128449−SRR24128469). FPKM (the number of fragments per kilobase transcript per million fragment map) was used to calculate the gene expression level. Genes with |log2FC|≥1 and Benjamini−Hochberg-adjusted P-values<0.05 were determined as differentially expressed genes (DEGs). Using the following databases for gene functional annotation: NCBI non-redundant protein sequences (Nr), Kyoto Encyclopedia of Genes and Genomes (KEGG), NCBI non-redundant nucleotide sequences (Nt), Gene Ontology (GO), a manually annotated and reviewed protein sequence database (Swiss-Prot), a manually annotated and reviewed protein sequence database (Pfam), and Clusters of Orthologous Groups of Proteins (KOG/COG).

Based on the results of gene functional annotation, the candidate key genes *PAL*, *C4H*, *4CL*, *CHS*, *CHI*, *F6OMT*, and *UGT* were selected in the homoplantaginin biosynthesis pathway. The sequences of *FNS* and *F6H* were obtained by a local BLAST algorithm-based search and relevant literature (Chen et al., 2014; Zhao et al., 2018). Phylogenetic tree construction with MEGA11 (https://megasoftware.net/). The Neighbor-Joining method was used to construct this tree with bootstrap (n=1000).

### *In vitro* functional analysis of candidate genes

The open reading frames (ORFs) of *PAL*, *4CL*, *CHS, CHI*, and *F6OMT*; *C4H*, *FNS*, and *F6H*; and *UGT* were individually cloned into the prokaryotic expression vectors pET28a, pET32a, and pSJ8-MBP, respectively. The primers used for construction are shown in **Supplementary Material** **Table S1**. Then, the recombinant plasmids were transferred into *E. coli* BL21(DE3), respectively. The transformants were grown in 100 mL Luria-Bertani (LB) medium containing antibiotics, which was cultured at 37°C. To express of target proteins, 0.5 mM IPTG (isopropyl 1-*β*-D-thiogalactoside) was added to the medium at OD_600_ 0.6–0.8 of the strains, and then further incubated at 16°C or 25°C for 16 h. The cells were collected by centrifugation, then re-suspended with phosphate-buffered saline, and disrupted using a sonicator on an ice-bath. The crude lysate was centrifuged (12000 rpm, 30 min) at 4°C, removed from the cell fragments. The supernatant was purified using Ni-NTA columns (Smart-Lifesciences, Changzhou, China) and further purified using protein purification system SDL-030-F2 (SePure Instruments Co., Ltd, Suzhou, China). The concentrations of protein were determined with the Bradford method. The purity of purified protein was detected by SDS-PAGE

The reaction volume was 200 µL. *SpPAL* reaction components included 100 mM Tris-HCl, 3 mM phenylalanine, and purified *SpPAL* enzyme, which were incubated at 37°C for 8 h. *SpC4H* reaction components included 100 mM Tris-HCl, 1 mM NADPH, 0.5 mM GSH (reduced glutathione), 50 μM cinnamic acid, and purified *SpC4H* enzyme, which were incubated at 28°C for 12 h. *Sp4CL* reaction components included 100 mM Tris-HCl, 5 mM ATP, 0.3 mM *p*-Coumaroyl-CoA, 5 mM MgCl_2_, 50 μM cumaric acid, and purified *Sp4CL* enzyme, which were incubated at 37°C for 6 h. *SpCHS* reaction components included 100 mM Tris-HCl, 5 mM ATP, 6 mM *p*-Coumaroyl-CoA, 5 mM MgCl_2_, 3 mM tyrosine, and purified *SpCHS* enzyme, which were incubated at 30°C for 12 h. *SpCHI* reaction components included 100 mM PBS, 50 μM naringin chalcone, and purified *SpCHI* enzyme, which were incubated at 25°C for 2 s. *SpFNS* reaction components included 100 mM Tris-HCl, 50 μM naringenin, 1 mM NADPH, 0.5 mM GSH, and purified *SpFNS* enzyme, which were incubated at 28°C for 12 h. *SpF6H* reaction components included 100 mM Tris-HCl, 50 μM apigenin, 0.5 mM GSH, 1 mM NADPH, and purified *SpF6H* enzyme, incubated at 28°C for 12 h. *SpF6OMT* reaction components included 100 mM Tris-HCl, 50 μM scutellarein, 1 mM SAM (S-adenosyl-L-methionine), 1 mM DTT (dithiothreitol), 1 mM MgCl_2_, and purified *SpF6OMT* enzyme, which were incubated at 28°C for 12 h. *SpUGT* reaction components included 50 mM PBS, 100 µM hispidulin, 1 mM UDPG (uridine diphosphate glucose), and purified *SpUGT* enzyme, which were incubation at 30°C for 4 h. Enzyme activity was analyzed *via* HPLC and UPLC-Q-TOF-MS/MS.

### Quantitative real-time PCR analysis

Total RNA from leaves, roots, stems, and flowers of *S. plebeia* was extracted as the protocol described above. Reverse transcription was done by using HiScript II Q RT SuperMix for qPCR (Code: R223-1, Vazyme, Nanjing, China). The ChemQ SYBR qPCR Master Mix (Code: 341-02, Vazyme, Nanjing, China) was used for qRT-PCR analysis and gene-specific primer pairs (**Table S2**). The relative expression levels of the target genes were evaluated using the 2^-△△Ct^ approach with Sp*β*-actin as the reference gene (Livak and Schmittgen, 2001). Each sample was analyzed in three biological replicates.

### Reconstitution of hispidulin and homoplantaginin biosynthesis pathway in bacterial and product analysis

The genes of homoplantaginin pathway with known function (*SpFNS*, *SpF6H1*, *SpF6OMT2*, and *SpUGT1*) were inserted in different combinations into the vectors pET32a and pACYCDuet-1 downstream of T7 promoters. The 3′ end of *SpFNS* and *SpF6H1* were fused to the 5′ end of *AtCPR via* the linker sequence ACTAGTGGTTCTACCTCTTCTGGTTCTGGT. The 5′ end of *SpUGT1* connects the MBP sequence of pSJ8-MBP vector. All the recombinant *E. coli* strains used in this study are shown in **Table S3**. The recombinant plasmids were transformed into *E. coli* after sequencing verified. The recombinant strains were cultured in LB medium with antibiotics at 37°C and 200 rpm until the OD_600_ reached 0.6-0.8. Then, 0.5 mM IPTG and 100 μM naringenin were added and cultured at 20°C and 135 rpm. After 24 h, 10 mL fermentation liquor was taken and mixed with a double volume of ethyl acetate to extract the reactions. The ethyl acetate layer was dried under reduced pressure, and dissolved with 100 μL methanol for HPLC analysis.

## Results

### Chemical profiling of different tissues of *S. plebeia* at developmental stages

**Figure 2:**
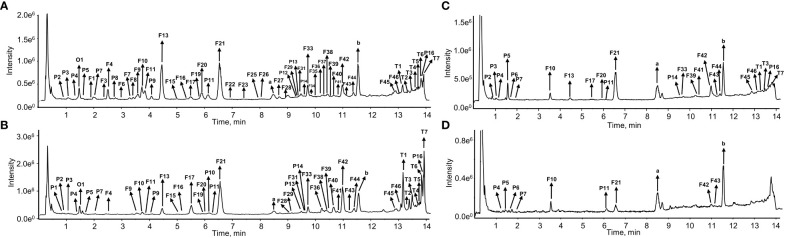
The total ion chromatograms (TIC) of *S. plebeia* at the flower stage in negative ion mode. **(A)** TIC of methanol extracts from leaves. **(B)** TIC of methanol extracts from flowers. **(C)** TIC of methanol extracts from stems. **(D)** TIC of methanol extracts from roots.

**Figure 3:**
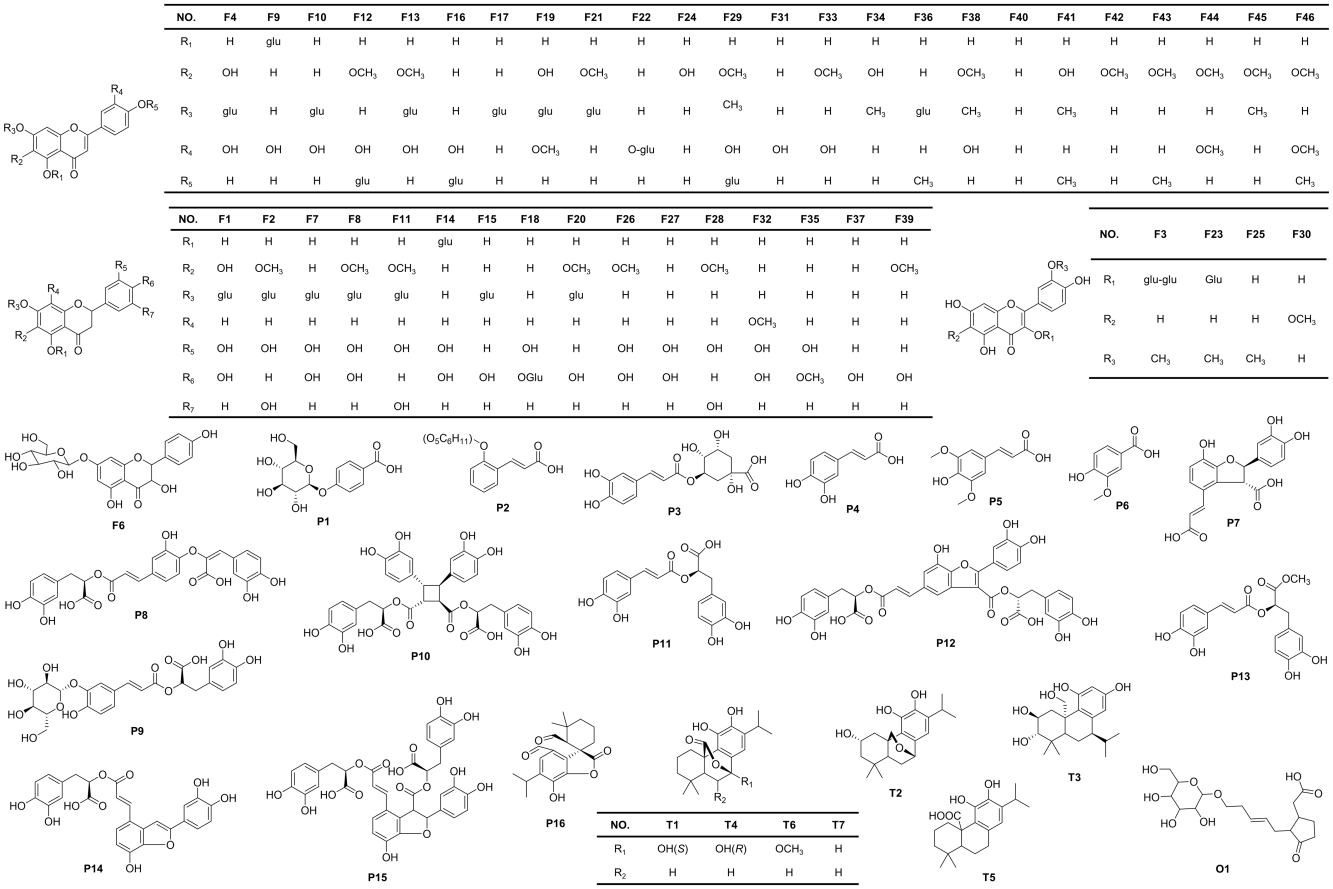
Chemical structures of the compounds identified in *S. plebeia*.

**Figure 4:**
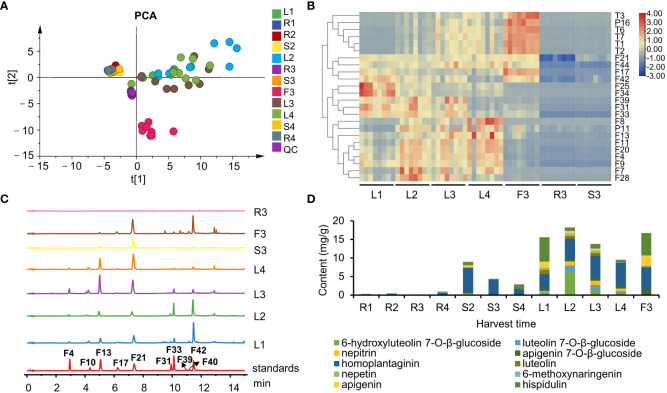
The chemical profiles of *S. plebeia* with different tissues and developmental stages. **(A)** PCA score plot for *S. plebeia* at different tissues and developmental stages based on 70 identified compounds. **(B)** Heatmap of DEMs in *S. plebeia* at different tissues and developmental stages. **(C)** HPLC chromatograms of *S. plebeia* samples at 342 nm. (F4: 6-hydroxyluteolin 7-*O*-β-glucoside, F10: luteolin 7-*O*-β-glucoside, F13: nepitrin, F17: apigenin 7-*O*-β-glucoside, F21: homoplantaginin, F31: luteolin, F33: nepetin, F39: 6-methoxynaringenin, F40: apigenin, F42: hispidulin). **(D)** The contents of the 10 main flavonoids in *S. plebeia*.

The chemical compositions of different organs of *S. plebeia* during growth and development were analyzed using UPLC-Q-TOF-MS/MS method. The total ion chromatograms (TICs) of different organs and developmental stages of *S. plebeia* are shown in **Figures 2**, **S1**. Combined with information such as the parent ion, molecular formula, retention time, secondary fragmentation, and relevant literature, a total of 70 compounds including 46 flavonoids, 16 phenolic acids, seven terpenoids, and one organic acid, were accurately or tentatively identified (**Table 1**; **Figure 3**). We identified 46 flavonoids, including 24 flavones, 17 flavonones, 4 flavanones and one flavanonol, of which 21 compounds were discovered in *S. plebeia* for the first time (**Table 1**). Especially, the peaks F2 and F5 were tentatively identified as new flavanone diglycosides from *S. plebeia*. The characteristic product ions and neutral losses are shown in the **Figure S2**. From the TICs and Venn graph (**Figures 2**, **S3**), we can see that there was small difference in the number of the identified compounds in the samples of different developmental stages, which 43 compounds were common. However, the number of the identified compounds in different organs varies greatly. For instance, 68 compounds were identified in the leaf, while only 21 compounds were identified in the root. Principal component analysis (PCA) also revealed that the stems and roots, flowers, and leaves clustered into three groups (**Figure 4A**). The distribution of compounds in different organs of *S. plebeia* is quite different. There was little difference in the compounds present in the roots and stems at different stages. Therefore, we selected leaves from different periods and different organs from the flowering stage for differential metabolite analysis. The groups showed clear separation in the OPLS-DA score plots of *S. plebeia* with satisfactory goodness of fit and statistical significance (**Figure S4**). Twenty-four of the 70 annotated metabolites were found to be differential metabolites (DEMs) (**Table S4**). Based on the relative contents of the DEMs in the different parts at different developmental stages, a heatmap was created (**Figure 4B**) and revealed differences. The compounds were mainly distributed in leaves and flowers and less existed in the stems and roots. Specifically, the flavonoids were the main DEMs and mainly distributed in the leaves, while terpenoids mostly existed in the flowers. Besides, the flavonoid metabolites in different developmental stages showed differences. The main DEMs were flavonoid aglycones in L1, and glycosides in L2, L3, and L4.

**Table 1:** Characterization of chemical constituents of *S. plebeia* by UPLC-Q-TOF-MS/MS.

No.	t_R_ (min)	Quasi-molecular (Error, ppm) [M-H]^−^	Molecular formula	*m/z* Calculated	MS/MS Fragments	Identification	Source
P1*	0.926	299.0764 (-2.8)	C_13_H_16_O_8_	299.0772	137	4-hydroxybenzoic acid 4-*O*-glucoside	F
P2*	1.058	325.0921 (-2.4)	C_15_H_18_O_3_	325.0929	163, 119	coumaric acid-*O*-hexoside	F, L (1,2,3,4), S (2,3,4)
P3*	1.077	353.0866 (-3.4)	C_16_H_18_O_9_	353.0878	191, 179, 161, 135	chlorogenic acid	F, L (1,2,3,4), S (2,3,4)
P4*	1.391	179.0358 (4.6)	C_9_H_8_O_4_	179.0350	135	caffeic acid	F, L (1,2,3,4), S (2,3,4), R (1,2,3,4)
O1*	1.471	387.1644 (-4.3)	C_18_H_28_O_9_	387.1661	207, 163, 119	tuberonic acid-glucoside	F, L (1,2,3,4)
P5*	1.576	223.0622 (4.5)	C_11_H_12_O_5_	223.0612	163, 135, 119	sinapic acid	F, L (2,3), S (2,3,4), R (1,2,3,4)
P6*	1.708	167.0356 (3.7)	C_8_H_8_O_4_	167.0350	123	vanillic acid	S (2,3,4), R (1,2,3,4)
F1*	1.987	465.1021 (-3.8)	C_21_H_22_O_12_	465.1039	303, 285, 181, 167, 153, 135	5,6,7,3’,4’-pentahydroxyflavanon 7-*O*-glucoronide	L (2,3,4)
P7*	2.109	357.0628 (3.4)	C_18_H_14_O_8_	357.0616	135, 109	przewalskinic acid A	F, L (1,2,3,4), S (2,3,4), R (1,2,3,4)
F2*	2.162	641.1716 (-1.1)	C_28_H_34_O_17_	641.1723	479, 317, 302, 181	5,7,3’,4’-tetrahydroxy-6-methoxy-flavanone glucobiose isomer 1	L (2,4)
F3*	2.371	639.1546 (-3.2)	C_28_H_32_O_17_	639.1567	477, 315, 300	isorhamnetin 3-*O*-gentiobioside	L (1,2,3)
F4	2.51	463.0872 (-2.2)	C_21_H_20_O_12_	463.0882	301, 300, 272, 255, 228, 137	6-hydroxyluteolin 7-*O*-β-glucoside	F, L (1,2,3,4), S (2,4)
F5*	2.787	641.1726 (0.4)	C_28_H_34_O_17_	641.1723	479, 317, 181	5,7,3’,4’-tetrahydroxy-6-methoxy-flavanone glucobiose isomer 2	L (2,4)
P8*	2.843	537.1075 (-4.1)	C_27_H_22_O_12_	537.1039	295, 269, 179, 135	salvianolic acid I	L (2,3)
F6*	3.067	449.1089 (-0.1)	C_21_H_22_O_11_	449.1089	287, 259, 181, 167, 153, 139, 119	dihydrokaempferol 7-*O*-glucoside	L (2,3,4)
F7*	3.322	449.1114 (5.5)	C_21_H_22_O_11_	449.1089	287, 151, 135, 107	eriodictyol-7-*O*-glucoside	L (1,2,3,4)
F8	3.42	479.1178 (-3.5)	C_22_H_24_O_12_	479.1195	317, 302, 284, 215, 181, 165, 135	5,7,3’,4’ -tetrahydroxy-6-methoxyflavanone-7-glucoside	L (1,2,3,4)
F9	3.588	447.0915 (-4.0)	C_21_H_20_O_11_	447.0933	285, 284, 283, 256, 228, 227, 217, 137	luteolin-5-*O*-*β*-D-glucoside	F, L (1,2,3,4), S (2,4)
F10	3.738	447.0920 (-2.9)	C_21_H_20_O_11_	447.0933	285, 284, 267, 257, 243, 241, 223, 217, 199, 151, 149, 147, 133, 107	luteolin 7-*O*-β-glucoside	F, L (1,2,3,4), S (2,3,4)
F11	3.86	479.1218 (-4.8)	C_22_H_24_O_12_	479.1195	317, 302, 181, 166, 135	3’,5’,5,7-tetrahydroxy-6-methoxy-7-*O*-β-D-glucoseflavanone	F, L (1,2,3,4), S (2,4)
P9*	4.105	521.1313 (2.4)	C_24_H_26_O_13_	521.1301	323, 197, 179, 161, 135	salviaflaside	F, L (2,3,4), S (2,4), R (1,2,4)
F12*	4.341	477.1018 (-4.3)	C_22_H_22_O_12_	477.1039	315, 313, 300, 299, 285, 271, 199, 159, 133	nepetin 4’-O-β-D-glucopyranoside	L (1), S (2,4)
F13	4.45	477.1020 (-3.9)	C_22_H_22_O_12_	477.1039	315, 314, 313, 300, 299, 285, 243, 227, 199, 133	nepitrin	F, L (1,2,3,4), S (2,3,4), R (1,2,4)
F14*	4.651	449.1084 (-1.2)	C_21_H_22_O_11_	449.1089	287, 151, 135	eriodictyol 5-​*O*-​β-​D-​glucoside	L (2,4)
F15*	5.019	433.1122 (-4.2)	C_21_H_22_O_10_	433.1140	271, 177, 151, 131, 119	naringenin-7-*O*-glucoside	F, L (1,2,3,4)
F16*	5.384	447.0919 (-3.1)	C_21_H_20_O_11_	447.0933	285, 284, 261, 241, 217, 185, 175, 151	luteolin-4’-*O*-β-D-glucoside	F, L (1,2,3,4)
F17	5.538	431.0999 (3.5)	C_21_H_20_O_10_	431.0984	269, 268, 151, 123, 117	apigenin 7-*O*-β-glucoside	F, L (1,2,3,4), S (2,3,4), R (4)
F18*	5.657	449.1081 (-1.9)	C_21_H_22_O_11_	449.1089	287, 151, 135, 125, 107	eriodictyol-4’-*O*-glucoside	L (2,4)
F19*	5.766	477.1020 (-3.9)	C_22_H_22_O_12_	477.1039	315, 300, 216, 200, 137	6-hydroxychrysoeriol-7-*O*-glucosided	F, L (1,2,3,4)
F20	5.898	463.1232 (-3.0)	C_22_H_24_O_11_	463.1246	301, 286, 285, 181, 166, 119	5,7,4’-trihydroxy-6-methoxy-flavanone-7-*O*-β-D-glucoside	F, L (1,2,3,4), S (2,3,4)
P10	6.135	719.1623 (0.8)	C_36_H_32_O_16_	719.1618	359, 197, 179, 161	sagerinic acid	F, L (2,3,4), S (4), R (1,2,4)
P11	6.162	359.0765 (-2.1)	C_18_H_16_O_8_	359.0772	197, 179, 161, 135, 132	rosmarinic acid	F, L (1,2,3,4), S (2,3,4), R (1,2,3,4)
F21	6.594	461.1072 (-3.8)	C_22_H_22_O_11_	461.1089	299, 298, 283, 269, 255	homoplantaginin	F, L (1,2,3,4), S (2,3,4), R (1,2,3,4)
F22*	6.826	447.0916 (-3.8)	C_21_H_20_O_11_	447.0933	285, 256, 241, 217, 175	luteolin-3’-*O*-β-D-glucoside	L (1,2,3,4)
F23*	7.475	477.1015 (-4.9)	C_22_H_22_O_12_	477.1039	315, 300, 299, 271	isorhamnetin-3-O-glucoside	L (1,2,3)
F24	7.581	285.0401(-1.3)	C_15_H_10_O_6_	285.0405	166, 139, 117	scutellarein	L (2)
F25	8.103	315.0494 (-5.2)	C_16_H_12_O_7_	315.0510	300, 299, 255, 227, 201, 137, 134	isorhamnetin	L (1,2,3,4), S (2,4)
F26	8.308	317.0651 (-5.0)	C_16_H_14_O_7_	317.0667	165, 135, 110	6-Methoxyeriodictyol	L (2,3,4)
F27	8.719	287.0552 (-3.2)	C_15_H_12_O_6_	287.0561	151, 135, 107	eriodictyol	L (1,2,3,4)
F28	9.008	317.0654 (-4.0)	C_16_H_14_O_7_	317.0667	302, 181, 166, 167, 152, 139, 135, 124	3’,5’,5,7-tetrahydroxy-6-methoxy-flavanone	F, L (1,2,3,4), S (2)
P12*	9.17	715.1309 (0.6)	C_36_H_28_O_16_	715.1305	337	schizotenuin A	L (3), S (2), R (1,2,4)
F29*	9.37	491.1200 (1.0)	C_23_H_24_O_12_	491.1195	329, 314, 299, 285	cirsiliol 4’-glucoside	F, L (3,4)
F30*	9.428	331.0448 (-3.4)	C_16_H_12_O_8_	331.0459	316, 271, 181, 166, 121	6-methoxyquercetin	L (2,4)
P13*	9.445	373.0934 (1.4)	C_19_H_18_O_8_	373.0929	197, 179, 175, 135	rosmarinic acid methyl ester	F, L (2,3), S (2,4), R (1,2,4)
F31	9.488	285.0415 (3.6)	C_15_H_10_O_6_	285.0405	267, 229, 217, 199, 175	luteolin	F, L (1,2,3,4), S (2)
F32*	9.507	317.0657 (-3.1)	C_16_H_14_O_7_	317.0667	166, 135, 124, 110	8-methoxyeriodictyol	L (2)
P14*	9.623	491.1001 (3.5)	C_26_H_20_O_10_	491.0984	311, 267, 135	salvianolic acid C	F, L (2,3,4), S (2,3,4), R (1,2,4)
F33	9.723	315.0525 (4.7)	C_16_H_12_O_7_	315.0510	300, 299, 255, 227, 201, 137, 133, 119	nepetin	F, L (1,2,3,4), S (2,3,4), R (2,4)
F34	9.845	299.0547 (-4.7)	C_16_H_12_O6	299.0516	284, 283, 255, 228, 227	7-*O*-Methylscutellarein	F, L (1,2,3,4), S (2,4)
F35*	9.991	301.0708 (-3.2)	C_16_H_14_O_6_	301.0718	230, 185, 152	hesperitin	L (2,3,4)
F36*	10.129	491.1219 (4.9)	C_22_H_22_O_10_	491.1195	311, 283, 168	acacetin-​7-​*O*-​glucoside	F, R (1,2,4)
F37	10.301	271.0601 (-4.0)	C_15_H_12_O_5_	271.0612	151, 119	naringenin	L (1,2,3)
F38*	10.347	329.0658 (-2.7)	C_17_H_14_O_7_	329.0667	299, 271, 243, 199, 171, 133	cirsiliol	F, L (1,2,3,4), S (4)
F39	10.452	301.0714 (1.27)	C_16_H_14_O_6_	301.0718	286, 229, 180, 165, 139, 139, 119	6-methoxynaringenin	F, L (1,2,3,4), S (2,3), R (2,4)
F40	10.664	269.0443 (-4.6)	C_15_H_10_O_5_	269.0455	241, 227, 225, 201, 183	apigenin	F, L (1,2,3,4), S (2)
F41	10.688	313.0719 (0.4)	C_17_H_14_O_6_	313.0718	161, 151, 133, 123	5, 6-dihydroxy-7, 4′-dimethoxyflavone	F, L (2,3,4), S (2,3,4), R (1,2,4)
P15*	10.839	717.1496 (4.9)	C_36_H_30_O_16_	717.1461	519, 339	salvianolic acid B	L (2,3), S (2,4), R (1,4)
F42	10.986	299.0562 (0.3)	C_16_H_12_O_6_	299.0561	284, 283, 255, 228, 227, 183, 164, 137, 117	hispidulin	F, L (1,2,3,4), S (2,3,4), R (1,2,3,4)
F43	11.3	313.0716 (-0.5)	C_17_H_14_O_6_	313.0718	300, 242, 226, 161, 133	pectolinarigenin	F, L (1,2,3,4), S (2,3,4), R (1,2,3,4)
F44*	11.374	329.0653 (-4.2)	C_17_H_14_O_7_	329.0667	314, 299, 271, 243, 227, 199	jaceosidin	F, L (1,2,3,4), S (2,3,4)
F45	12.915	313.0698 (-6.3)	C_17_H_14_O_6_	313.0718	297, 283, 255, 227, 163	cirsimaritin	F, L (1,2,3,4), S (2,3,4)
F46	13.134	343.0811 (-3.6)	C_18_H_16_O_7_	343.0823	328, 313, 298, 285, 270, 214, 198	eupatilin (5,7-Dihydroxy-3′,4′,6-trimethoxyflavone)	F, L (1,2,3,4), S (2,3)
T1	13.162	345.1709 (0.4)	C_20_H_26_O_5_	345.1707	301, 283, 268, 253	rosmanol	F, L (2,3,4), S (2,3,4)
T2	13.174	331.1913 (0.6)	C_20_H_28_O_4_	331.1915	301, 283, 267, 241, 215, 201	2,​11,​12-​Trihydroxy-​7,​20-​epoxy-​8,​11,​13-​abietatriene	F, L (2,3,4), S (4)
T3	13.392	349.2025 (1.3)	C_20_H_30_O_5_	349.2020	331, 319, 301, 283, 267	plebeianiol A	F, L (2,3,4), S (2,3,4)
T4	13.717	345.1717 (2.8)	C_20_H_26_O_5_	345.1707	301	epirosmanol	F, L (2,3)
T5*	13.774	331.1923 (2.5)	C_20_H_28_O_4_	331.1915	301, 285, 203	carnosic acid	F, L (2,3,4), S (2,4)
T6*	13.837	359.1874 (2.8)	C_21_H_28_O_5_	359.1864	283, 268, 267, 227	epirosmanol methyl ether	F, L (2,3,4), S (2,3,4)
P16	13.849	343.1565 (4.1)	C_20_H_24_O_5_	343.1551	299, 243, 216	rosmadial	F, L (2,3,4), S (2,3,4)
T7	13.932	329.1759 (0.2)	C_20_H_26_O_4_	329.1758	285, 201	carnosol	F, L (2,3,4), S (2,3,4)

*These components were discovered in *S. plebeia* for the first time in this study.

To further accurately explore the contents of the main flavonoids in different parts at different plant developmental stages, 10 flavonoids (6-hydroxyluteolin 7-*O*-β-glucoside, nepitrin, luteolin 7-*O*-β-glucoside, apigenin 7-*O*-β-glucoside, homoplantaginin, luteolin, nepetin, 6-methoxynaringenin, apigenin, and hispidulin) were quantitatively analyzed using HPLC-DAD. Results indicated that flavonoid content varied among different organs and at different growth periods of the plant (**Figures 4C, D**). Regarding the distribution of flavonoids among different organs, the flavonoids were mainly distributed in leaves and flowers and to a less extent in the stems and roots. In different growth and development periods of the plant, flavonoid content peaked at the stem elongation stage. Specifically, flavonoid content in the leaves followed a parabolic trend and peaked when the stem elongated (L2, 18.21 ± 0.33 mg/g). Similarly, the flavonoid content in the stems was also the highest in S2 (8.97 ± 0.25 mg/g). In addition, the flavonoid content in flowers was up to 16.73 ± 0.16 mg/g (F3). A fluctuating trend of increase, decrease, and increase trend was observed in the roots; however, the highest yield was only 0.94 ± 0.08 mg/g at the ripening stage (R4).

Notably, hispidulin and homoplantaginin exhibited the highest levels in all the organs at each developmental stages of the plant. Our results showed that they accumulated differently in different growth stages (**Figure S5**). The content of hispidulin reached the highest level of 6.55 ± 1.44 mg/g in the leaves at the basal leaf stage, and dropped to about 1 mg/g from vegetative growth to reproductive stage, but remained abundant in flowers (6.07 ± 0.43 mg/g). Homoplantaginin content in leaves gradually increased from 3.63 ± 0.59 to 6.38 ± 0.68 mg/g from the basal leaf to the ripening stage. A similar trend was observed for accumulation of 6-hydroxyluteolin 7-*O*-β-glucoside, luteolin 7-*O*-β-glucoside, and nepitrin. As the glycosides are maybe more stable than aglycones in the plant (Bowles et al., 2005), aglycones might transform into glycosides during the accumulation process. The homoplantaginin content in the stem was high in the vegetative growth period, but decreased during the reproductive stage. In addition, similar to hispidulin, homoplantaginin was abundant in flowers (5.73 ± 0.59 mg/g).

### Transcriptomic analysis of different organs and developmental stages of *S. plebeia*

To further study the mechanisms of flavonoid biosynthesis in different organs and developmental stages of *S. plebeia*, 21 samples were used for transcriptomic analysis. The analysis produced approximately 161.44 Gb of clean data and 58,905 unigenes (140,509 transcripts) with a mean length of 1161.286 nt and an N50 of 1754 nt, following *de novo* assembly. The Q30 and GC percentages ranged from 94.46% to 95.15% and from 47.3% to 49.96%, respectively (**Tables S5, S6**). The KEGG, NR, GO, Swissprot, KOG, COG, TrEMBL, Pfam, and eggNOG databases were used to annotate the predicted protein sequences. Finally, 44,185 unigenes were functionally annotated (**Table S7**). Results from qPCR validation were mostly consistent with those obtained using RNA-seq, indicating the accuracy of the sequencing data (**Figure S6**). Accordingly, the RNA-seq data were used for further analysis.

**Figure 5:**
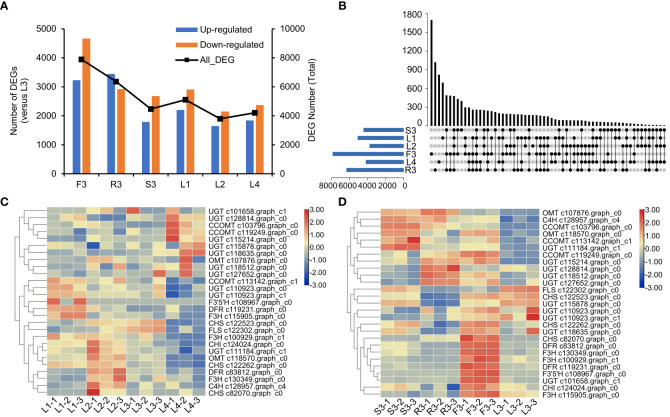
Differentially expressed genes (DEGs) of *S. plebeia* with different tissues and developmental stages. **(A)** The changes of upregulated DEGs and downregulated DEGs in different groups, with L3 as control groups. **(B)** Venn diagram for all DEGs in different groups, with L3 as control groups. **(C)** The heatmap was drawn based on relative expression of annotated flavonoid biosynthesis genes in KEGG pathway in different developmental stages. **(D)** The heatmap was drawn based on relative expression of annotated flavonoid biosynthesis genes in KEGG pathway in different developmental tissues.

We then screened the differentially expressed genes (DEGs) in different organs and developmental stages of *S. plebeia*. There were 7896 DEGs in F3, 6366 DEGs in R3, 4471 DEGs in S3, 5112 DEGs in L1, 3798 DEGs in L2, 4211 DEGs in L4 compared with those in L3 (**Figure 5A**; **Table S8**). The number of DEGs in different tissues was higher than that in the developmental stages, which were consistent with the trend of metabolite accumulation. The statistical significance of the differences in gene expression in *S. plebeia* in different tissues and developmental stages was also represented using the volcano plots (**Figure S7**). The number of DEGs varied in different groups, and there were more special DEGs in the flowers and roots (**Figure 5B**). DEGs were further assigned to the KEGG pathway. Interesting, the metabolites involved in “flavonoid biosynthesis” and “phenylpropanoid biosynthesis” were significantly enriched in different tissues and developmental stages (**Figure S8**).

Next, we focused on the genes involved in flavonoid biosynthesis. A total of 27 annotated flavonoid biosynthesis genes were screened from the differential transcript analysis. Based on their expression (**Table S9**), a heatmap were drawn (**Figures 5C, D**). We found that at different stages, most flavonoid biosynthesis genes, including *C4H* (c128957.graph_c4), *CHI* (c124024.graph_c0), *CHS* (c82070.graph_c0), *F3H* (c130349.graph_c0), and *DFR* (c83812.graph_c0), were upregulated in L2 (**Figure 5C**). Notably, *UGT* (c101658.graph_c1, c115214.graph_c0, c115878.graph_c0, c118512.graph_c0, c118635.graph_c0, c127652.graph_c0, and c128814.graph_c0) and *CCOMT* (c103796.graph_c0 and c119249.graph_c0) were upregulated in L4 (**Figure 5D**). Specifically, *UGT* and *CCOMT* are the key enzymes involved in the synthesis of homoplantaginin and hispidulin, respectively. This was consistent with the highest concentration of homoplantaginin observed at L4. At the flowering stage, the annotated flavonoid genes were highly expressed in flowers, such as *CHS* (c122262.graph_c0 and c82070.graph_c0), *DFR* (c119231.graph_c0 and c83812.graph_c0), *F3H* (c115905.graph_c0, c130349.graph_c0, and c100929.graph_c1), and *CHI* (c124024.graph_c0). Some OMT (c107876.graph_c0 and c118570.graph_c0), CCOMT (c103796.graph_c0, c119249.graph_c0, and c113142.graph_c1), and UGT (c115214.graph_c0, c118512.graph_c0, c127652.graph_c0, c128814.graph_c0, and c111184.graph_c1) were upregulated in S3 and R3.

### Identification and functional validation of candidate biosynthetic enzymes involved in hispidulin and homoplantaginin biosynthesis

**Figure 6:**
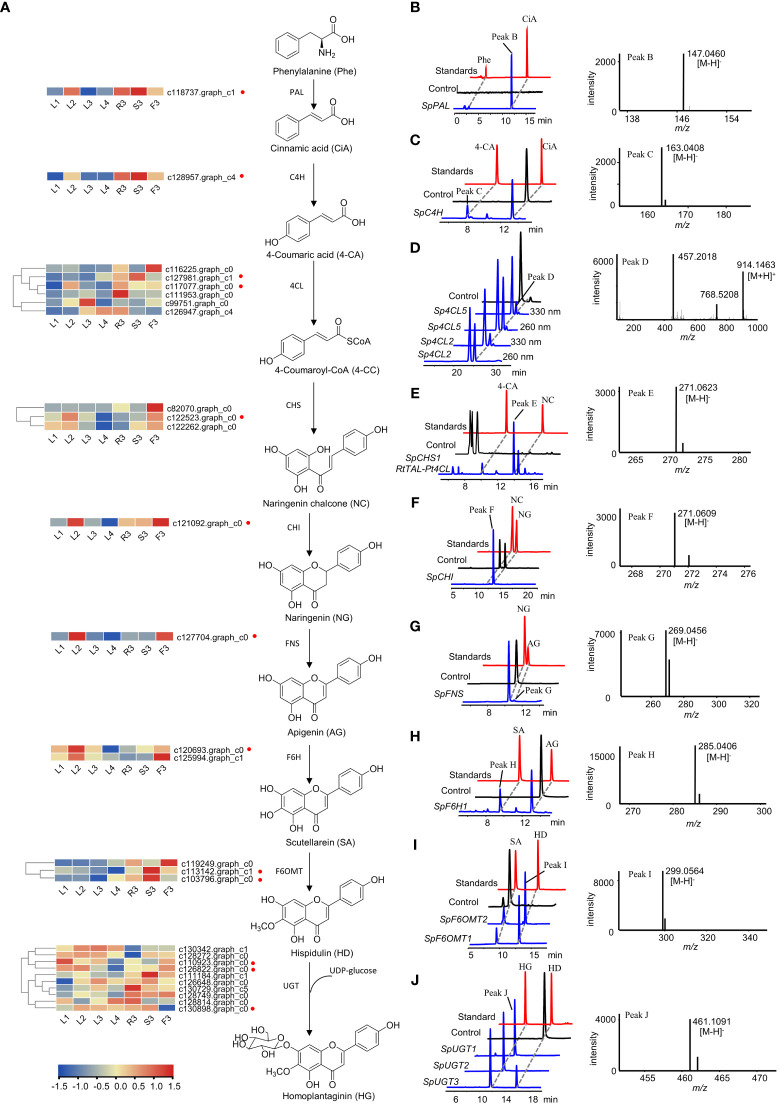
Functional verification of the candidate genes involved in homoplantaginin biosynthesis. **(A)** The candidate genes involved in homoplantaginin biosynthesis. The heatmap shows the relative expression levels of the candidate genes in different tissues and developmental stages of *S. plebeia*. **(B, D–F, I)** HPLC-DAD and UPLC-Q-TOF-MS/MS analysis of the *in vitro* enzymatic products of recombinant *SpPAL* (B, phenylalanine (Phe) as substrate), *Sp4CL2,5* (D, 4-coumaric acid (4-CA) as substrate), *SpCHS1* (E, 4-coumaric acid (4-CA) as substrate), *SpCHI* (F, naringenin chalcone (NC) as substrate), and *SpF6OMT1,2* (I, scutellarein (SA) as substrate) expressed in *E. coli* using pET28a as expression vector. **(C, G, H)** HPLC-DAD and UPLC-Q-TOF-MS/MS analysis of the *in vitro* enzymatic products of recombinant *SpC4H* (C, cinnamic acid (CiA) as substrate), *SpFNS* (G, naringenin (NG) as substrate), and *SpF6H1* (H, apigenin (AG) as substrate) expressed in *E. coli* using pET32a as expression vector. **(J)** HPLC-DAD and UPLC-Q-TOF-MS/MS analysis of the *in vitro* enzymatic products of recombinant *SpUGT1,2,3* (hispidulin (HD) as substrate) expressed in *E. coli* using pSJ8-MBP as expression vector. Samples identified in the work are shown in blue, standards are shown in red, and control (enzyme inactivation by boiling) are shown in black.

The biosynthetic pathways of flavonoids have been extensively studied in *Arabidopsis thaliana*, *Scutellaria baicalensis*, *Carthamus tinctorius* (Saito et al., 2013; Xu et al., 2020; Wang et al., 2021). Hence, the biosynthetic pathway from L-phenylalanine to homoplantaginin was predicted, comprising nine consecutive steps (**Figure 6A**). To fully screen for the possible unigenes involved in the biosynthesis of hispidulin and homoplantaginin, we mined the candidate genes by combining differential transcript analysis with transcriptome annotation. A total of 49 candidate genes, including two *PAL*, 17 *4CL*, two *C4H*, three *CHS*, two *CHI*, five *F6OMT*, and 18 *UGT*, were obtained. However, *FNS* and *F6H* were not annotated in the transcriptome analysis. Therefore, we searched for these two genes using the local BLAST and relevant literature. FNS has been reported to be divided into FNS I and FNS II. FNS I is mainly found in Umbelliferae, whereas FNS II is widely distributed in plants containing flavonoids. Therefore, we chose FNS I (GenBank: AY817680.1) from *Petroselinum crispum* (Umbelliferae) and FNS II (GenBank: KP337723.1) from *Salvia miltiorrhiza* (Labiatae) as templates to conduct a similarity search against the *S. plebeia* RNA-sequencing (RNA-Seq) datasets. However, only one gene (c127704.graph_c0) with high similarity to FNS II was found. The phylogenetic analysis demonstrates that *SpFNS* clustered with members of the FNS II clade and was closest in affinity to the CYP93B25 from *Salvia officinalis*, and FNS II from *Ziziphora clinopodioides* (**Figure S9**). *F6H* is mainly from the CYP71D family of Leguminosae and the CYP82D family of Labiatae. Considering the phylogenetic relationships among the species, the *F6H* of *S. plebeia* is more likely to have originated from the CYP82D family. Hence, c120693.graph_c0, c125994.graph_c1, and c127863.graph_c4 were annotated as CYP82D family in our datasets, which named *SpF6H1*, *SpF6H2*, and *SpF6H3*. These three genes cluster in family CYP82D with CYP82D1.1 from *Scutellaria baicalensis*, which has been shown to have broad catalytic activity for 6-hydroxyflavonoids such as apigenin (**Figure S10**).

Finally, 53 unigenes were selected as candidate genes (**Table S10**). The sequences of the 53 genes were used as the queries in a BLAST algorithm-based search of NCBI databases to predict their possible functions and the open reading frame (ORF) sequences. Finally, 28 candidate genes were selected to validate their catalytic functions (**Table S1**).

Specifically, *SpPAL*, *SpC4H*, *Sp4CL1-6*, *SpCHS1-3*, *SpCHI*, *SpFNS*, *SpF6H1-2*, *SpF6OMT1-3*, and *SpUGT1-10* were expressed in *E. coli*, respectively. The recombinant proteins were extracted and purified for biochemical analysis *in vitro*. HPLC-DAD and LC-MS analyses showed that Sp*PAL* can catalyze the formation of cinnamic acid from phenylalanine (**Figure 6B**). When *AtCPR* proteins and NADPH were added, *SpC4H* was able to catalyze the production of 4-coumaric acid from cinnamic acid (**Figure 6C**). As we do not have the standard of 4-coumaryl-CoA, we identified the reaction products of *Sp4CL2* and *Sp4CL5* by comparing the different peak areas of 4-coumaryl-CoA at 330 and 260 nm according to a previous report (**Figure 6D**) (Li et al, 2010). Similarly, in order to verify the enzymatic activity of *SpCHS1–3*, we used a binary plasmid with pCDFDuet-*RtTAL*-*Pt4CL* and pET28a*-SpCHS1–3* co-expressed in *E. coli*. The co-expression of *RtTAL* and *Pt4CL* can directly catalyze tyrosine into 4-coumaryl CoA, providing a sufficient substrate for the catalytic reaction of *SpCHS*. Upon feeding with tyrosine, naringenin chalcones were found in the culture extracts (**Figure 6E**). Although naringenin chalcone can be converted to naringenin spontaneously, it can be converted almost completely instantaneously in the presence of CHI. As shown in **Figure 6F**, some naringenin was also generated in the control group, but naringenin chalcone was completely consumed in the sample group to generate naringenin, indicating the catalytic activity of *SpCHI*. *FNS* and *F6H*, which belong to the CYP450 family and require the NADPH-CPR to provide electrons, are similar to *C4H*. *AtCPR* was co-expressed with *SpFNS* and *SpF6H1-2*, respectively. *SpFNS* produced apigenin from naringenin (**Figure 6G**). *SpF6H1* produced scutellarein from apigenin, a precursor of hispidulin (**Figure 6H**). *SpF6OMT1* and *SpF6OMT2* converted scutellarein into hispidulin (**Figure 6I**). Thus, *SpPAL*, *SpC4H*, *Sp4CL2*, *Sp4CL5*, *SpCHS1*, *SpCHI*, *SpFNS*, *SpF6H1*, *SpF6OMT1*, and *SpF6OMT2* formed a complete biosynthetic pathway for hispidulin. In the final step of homoplantaginin biosynthesis, *SpUGT1*, *SpUGT2*, and *SpUGT3* produced homoplantaginin from hispidulin *in vitro* using UDP-glucose as a sugar donor (**Figure 6J**). In sum, all the enzymes involved in the hispidulin and homoplantaginin biosynthetic pathways were identified.

### Heterologous production of hispidulin and homoplantaginin in *E. coli*

**Figure 7:**
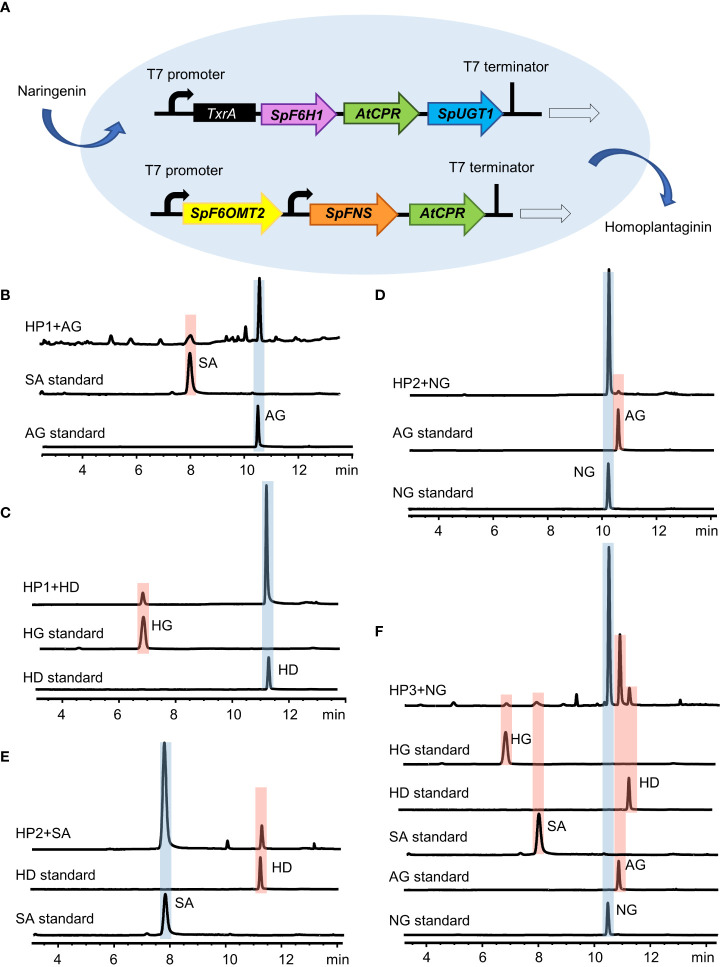
Reconstitution of the biosynthesis pathway of homoplantaginin in *E. coli*. **(A)** Schematic of the recombinant *E. coli* strain HP3. **(B)** HPLC analysis of apigenin (AG) and scutellarein (SA) standards and the fermented products of the recombinant *E. coli* strain HP1 with apigenin as a precursor. **(C)** HPLC analysis of hispidulin (HD) and homoplantaginin (HG) standards and the fermented products of the recombinant *E. coli* strain HP1 with hispidulin as a precursor. **(D)** HPLC analysis of naringenin (NG) and apigenin (AG) standards and the fermented products of the recombinant *E. coli* strain HP2 with naringenin as a precursor. **(E)** HPLC analysis of scutellarein (SA) and hispidulin (HD) standards and the fermented products of the recombinant *E. coli* strain HP2 with scutellarein as a precursor. **(F)** HPLC analysis of the activity of the recombinant *E. coli* strain HP3 by feeding naringenin (NG).

To further investigate the key enzymes involved in the hispidulin and homoplantaginin pathway in the *S. plebeia*, we reconstructed the pathway leading from naringenin to homoplantaginin in *E. coli* (**Figures 7A**). Simultaneously introduced *SpF6H1* and *SpUGT1* into *E. coli* to harbor *AtCPR*, the recombinant *E. coli* HP1 was able to produce scutellarein and homoplantaginin (**Figures 7B, C**). When *SpFNS* and *SpF6OMT2* were simultaneously introduced into *E. coli*, which harbored the *AtCPR*, the resulting recombinant *E. coli* HP2 was able to produce apigenin and hispidulin (**Figures 7D, E**). The pET32a vector carrying *SpF6H1* and *SpUGT1* and the pACYCDuet-1 vector carrying *SpFNS* and *SpF6OMT2* were transferred into *E. coli* to construct HP3. HP3 was able to produce hispidulin and homoplantaginin under naringenin induction. After 48 h of cultivation, the accumulation of hispidulin and homoplantaginin reached to 5.33 and 3.86 mg/L, respectively (**Figure 7F**). Although the yield was not desirable, it could be increased through metabolic engineering and fermentation optimization to make the full use of the *E. coli* fermentation system in the future.

## Discussion

Flavonoids were found to be the main components of *S. plebeia*, which are mainly present in leaves and flowers owing to their photosynthetic function and UV protection (Mathesius, 2018). Flavonoid levels vary during plant growth. During the basal leaf period in early March (stage 1), flavonoids levels in the leaves reached a peak during winter. As the plant matures, growth accelerates. The flavonoid content reached its maximum value in early April (stage 2). In early May, *S. pebeia* enters the reproductive stage. During the flowering stage (stage 3), the flavonoid content in the stems and leaves decreased to some extent, whereas the flavonoid content in flowers was as high as 16.73 ± 0.16 mg/g, which was higher than that in the leaves. This might be due to the fact that flavonoids are mostly synthesized in breeding organs for reproductive growth at the bloom stage, while synthesis of flavonoids in vegetative organs may be suppressed. The transcriptomic analysis showed that most of the genes involved in flavonoid synthesis were highly expressed in flowers (**Figure 5D**). At the later stage of reproduction (stage 4, early June), the flavonoid content in the stems and leaves decreased to its lowest. These findings suggest that late April (before the bloom stage) is the optimum harvest period for *S. plebeia.* Contrastingly, the contents of phenolic diterpenes, such as rosmanol and carnosol, were higher in flowers and leaves at the flowering stage (**Figure 4B**). Rosmanol and carnosol also possess anti-inflammatory and anti-tumor properties (Loussouarn et al., 2017; Jiang et al., 2021). Therefore, harvesting *S. plebeia* at the flowering stage is supported by pharmacopeial guidelines. Overall, determining the optimal harvest time for *S. plebeia* should be based on the desired chemical constituents and their intended use. If flavonoids are the main evaluation index, late April (before the bloom stage) is the optimum harvest time. If both flavonoids and terpenoids are in demand, May (the flower stage) is the optimum harvest time for *S. plebeia.*

We also characterized the complete biosynthetic pathway of hispidulin and homoplantaginin, which are main components of *S. plebeia*. The biosynthetic pathway from L-phenylalanine to scutellarein has been reported (Pei et al., 2022). *PaF6OMT* from the liverwort species *Plagiochasma appendiculatum* can convert scutellarein into hispidulin (Zhang et al., 2016). Although the key enzymes involved in hispidulin biosynthesis have been reported in other plant biosynthetic pathways, the complete pathway of hispidulin biosynthesis in *S. plebeia* has not been reported. Homoplantaginin is a 7-*O*-glycosylated hispidulin product. Glycosylation is commonly post-modified of flavonoids, which is beneficial to improve the stability and water solubility of flavonoids. Thus, it can help the regulation of bioactivity and the storage and detoxification of xenobiotics in plants (Bowles et al., 2005). In addition, glycosylation can be better absorbed by the human body compared to aglycones and has the potential to improve pharmacokinetic and pharmacodynamic profiles, making it better prospects for clinical use (Goel et al., 2021). While flavonoid 7-O-glycosyltransferase has been identified in many plants (Han et al., 2021; Pei et al., 2022), the UGT responsible for catalyzing hispidulin to homoplantaginin has not been reported. In this study, *SpUGT1*, *SpUGT2*, and *SpUGT3* were confirmed to be the flavonoid-7-*O*-glycosyltransferase. *SpUGT1*, *SpUGT2* and *SpUGT3* were submitted to the *UGT* Nomenclature Committee and designated as *UGT88A47*, *UGT88T1* and *UGT71AP4*, respectively. The UGT88 family was previously reported as a unique UGT family in Lamiales (Noguchi et al., 2009). Based on this, downstream genes were reconstructed in *E. coli* to enable biosynthesis of hispidulin and homoplantaginin using naringin as a precursor. However, biosynthesis was not initiated from tyrosine or glucose, and further work by synthetic biologists is necessary. Future measures to increase precursor supply, enhance cofactor levels, establish specific metabolic channels, and use co-culture technology will be taken.

## Conclusion

This study presents a comprehensive and systematic analysis of the chemical profile of various tissues of *S. plebeia* at different developmental stages, which is a novel contribution to the scientific literature. Furthermore, for the first time, we generated a transcriptome dataset for *S. plebeia* across different developmental stages and tissues. Through the integration of metabolomic-transcriptomic analysis and *in vitro* functional verification, we identified the enzymes involved in the biosynthetic pathways of hispidulin and homoplantaginin. In addition, we successfully reconstructed hispidulin and homoplantaginin in a heterologous host. These findings enhance our understanding of the distribution, accumulation, and biosynthesis mechanisms of flavonoids and provide a series of candidate genes for synthetic biology applications aimed at the production of natural products.

## Data availability statement

The datasets presented in this study can be found in online repositories. The names of the repository/repositories and accession number(s) can be found below: https://www.ncbi.nlm.nih.gov/, PRJNA952802, https://www.ncbi.nlm.nih.gov/, OQ786792 https://www.ncbi.nlm.nih.gov/, OQ786793, https://www.ncbi.nlm.nih.gov/, OQ786794, https://www.ncbi.nlm.nih.gov/, OQ786795, https://www.ncbi.nlm.nih.gov/, OQ786796, https://www.ncbi.nlm.nih.gov/, OQ786797, https://www.ncbi.nlm.nih.gov/, OQ786798, https://www.ncbi.nlm.nih.gov/, OQ786799, https://www.ncbi.nlm.nih.gov/, OQ786800, https://www.ncbi.nlm.nih.gov/, OQ786801, https://www.ncbi.nlm.nih.gov/, OQ786802, https://www.ncbi.nlm.nih.gov/, OQ786803, https://www.ncbi.nlm.nih.gov/, OQ786804.

## Author contributions

MQ and YuZ designed the research. YD, ZY collected plant material. YD, ZY, RZ, and HL performed the experiments. YD, SL, and LS analyzed the data. YD and MQ wrote the manuscript. YaZ, GX, and YuZ modified the language and revised the manuscript. All authors contributed to the article and approved the manuscript.
