# Supplementary material for: The chemical profiling of Salvia plebeia during different growth periods and the biosynthesis of its main flavonoids ingredients
Source: Front Plant Sci. 2023 Aug 14;14:1228356. doi: 10.3389/fpls.2023.1228356 (PMC10461478; doi:10.3389/fpls.2023.1228356)
Supplement: Supplementary file 1 [file DataSheet_1.pdf]

SUPPORTING INFORMATION for

The chemical profiling of *Salvia plebeia* during different growth periods and the biosynthesis of its main flavonoids ingredients

Yiqun Dai[1,2†], Ziyu Ye[1†], Hui Liu[3], Ruirui Zhu[1], Lanlan Sun[1], Shuai Li[1], Guoyong Xie[1], Yan Zhu[1], Yucheng Zhao[1*], Minjian Qin[1*]

[1] Department of Resources Science of Traditional Chinese Medicines, School of Traditional Chinese Pharmacy, China Pharmaceutical University, Nanjing 211198, China

[2] School of Pharmacy, Bengbu Medical College, 2600 Donghai Road, Bengbu 233030, China

[3] Yangzhou Center for Food and Drug Control, Yangzhou 225000, China

[†] These authors contributed equally to this study

* Corresponding author:

Dr. Yucheng Zhao: zhaoyucheng1986@126.com

Dr. Minjian Qin: minjianqin@163.com

Methods

HPLC analytical conditions for functional validation of candidate biosynthetic enzymes. An Agilent HPLC 1100 instrument was used (Agilent Technologies, Germany). Samples were separated on an Agilent Zorbax SB-C18 column (4.6×250 mm, 5 μm). The injection volume is 20 μL each time. The column temperature was 25 °C. The mobile phase consisted of 0.1% formic acid in water (v/v, A) and acetonitrile (B) at a flow rate of 1 ml/min. The gradient elution program for analyzing the products of PAL, CHS, F6H, F6OMT, and CHI was used: 0 min, 20% B, 10 min, 45% B, 20 min, 60% B, 25 min, 100% B. The gradient elution program for analyzing the products of C4H and FNS was used: 0 min, 30% B, 5 min, 40% B, 10 min, 50% B, 25 min, 60% B. The gradient elution program for analyzing the products of 4CL was used: 0 min, 5% B, 5 min, 5% B, 25 min, 25% B, 40 min, 30% B, 45 min, 100% B. The gradient elution program for analyzing the products of UGT was used: 0 min, 30% B, 15 min, 80% B, 17 min, 100% B, 20 min, 100% B.

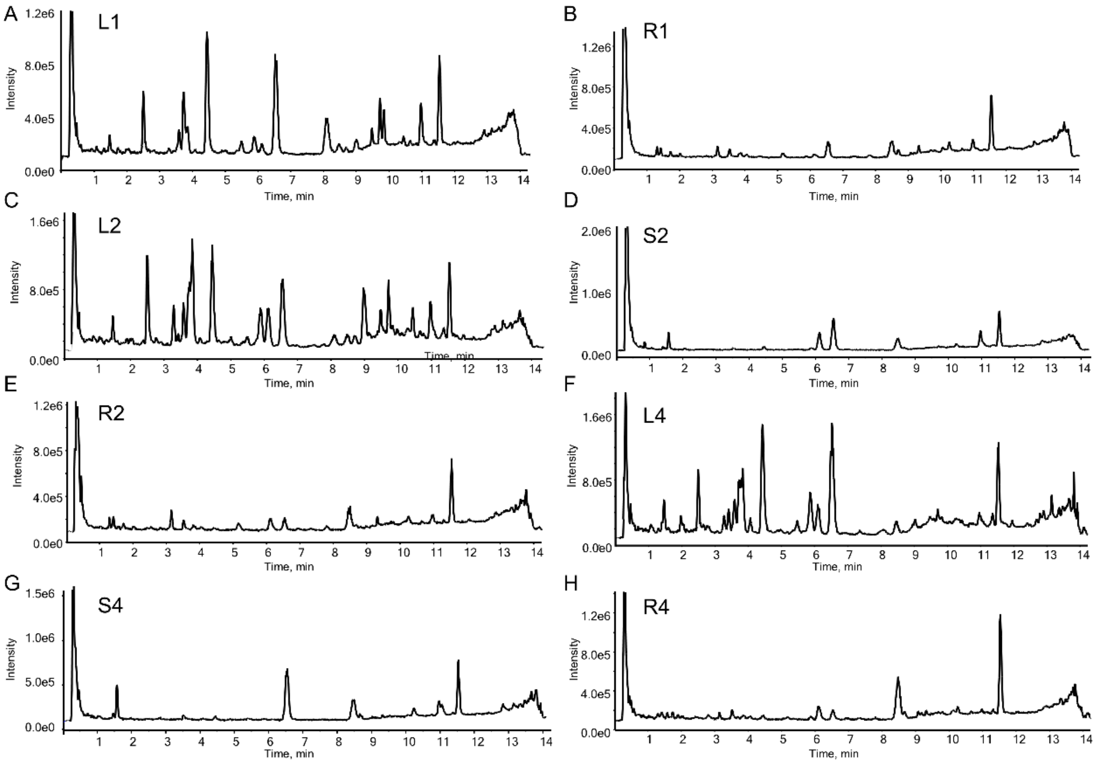

Figure S1. TIC of *S. plebeia* at basal leaf, stem elongation, and ripening stage in negative ion mode. (A) TIC of leaves methanol extract at basal leaf stage (L1) of *S. plebeia*. (B) TIC of roots methanol extract at basal leaf stage (R1) of *S. plebeia*. (C) TIC of leaves methanol extract at stem elongation stage (L2) of *S. plebeia*. (D) TIC of stems methanol extract at stem elongation stage (S2) of *S. plebeia*. (E) TIC of roots methanol extract at stem elongation stage (R2) of *S. plebeia*. (F) TIC of leaves methanol extract at ripening stage (L4) of *S. plebeia*. (G) TIC of stems methanol extract at ripening stage (S4) of *S. plebeia*. (H) TIC of roots methanol extract at ripening stage (R4) of *S. plebeia*.

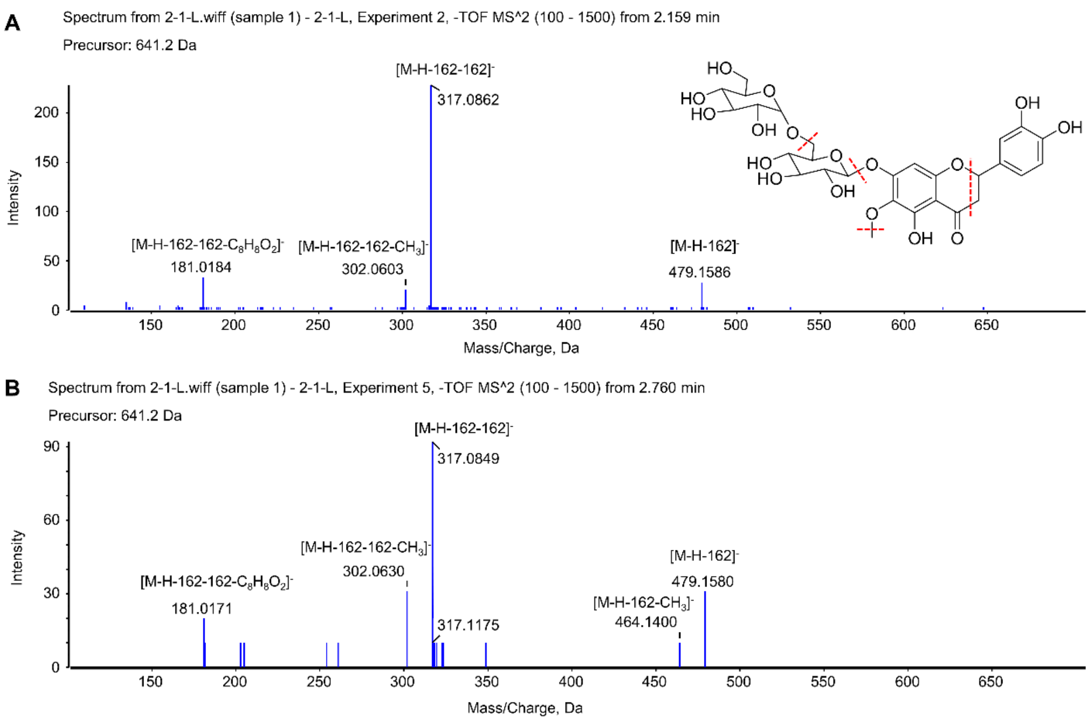

Figure S2. Negative ion ESI-MS^n spectra and the proposed fragmentation pathways of F2 and F5. (A) Negative ion ESI-MS^n spectra and the proposed fragmentation pathways of F2. (B) Negative ion ESI-MS^n spectra and the proposed fragmentation pathways of F5.

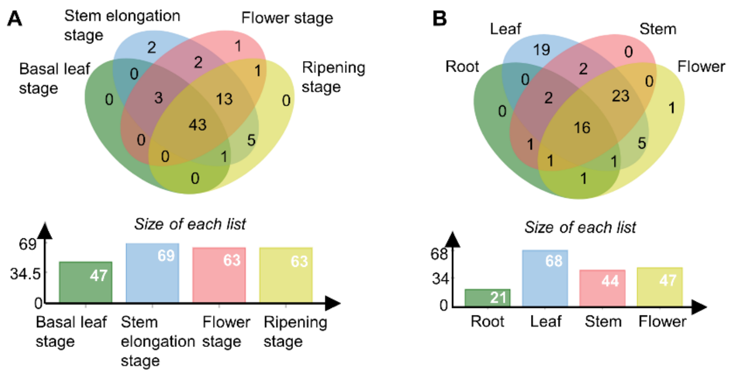

Figure S3. Venn diagrams of identified compounds in different organs and developmental stages of *S. plebeia*.

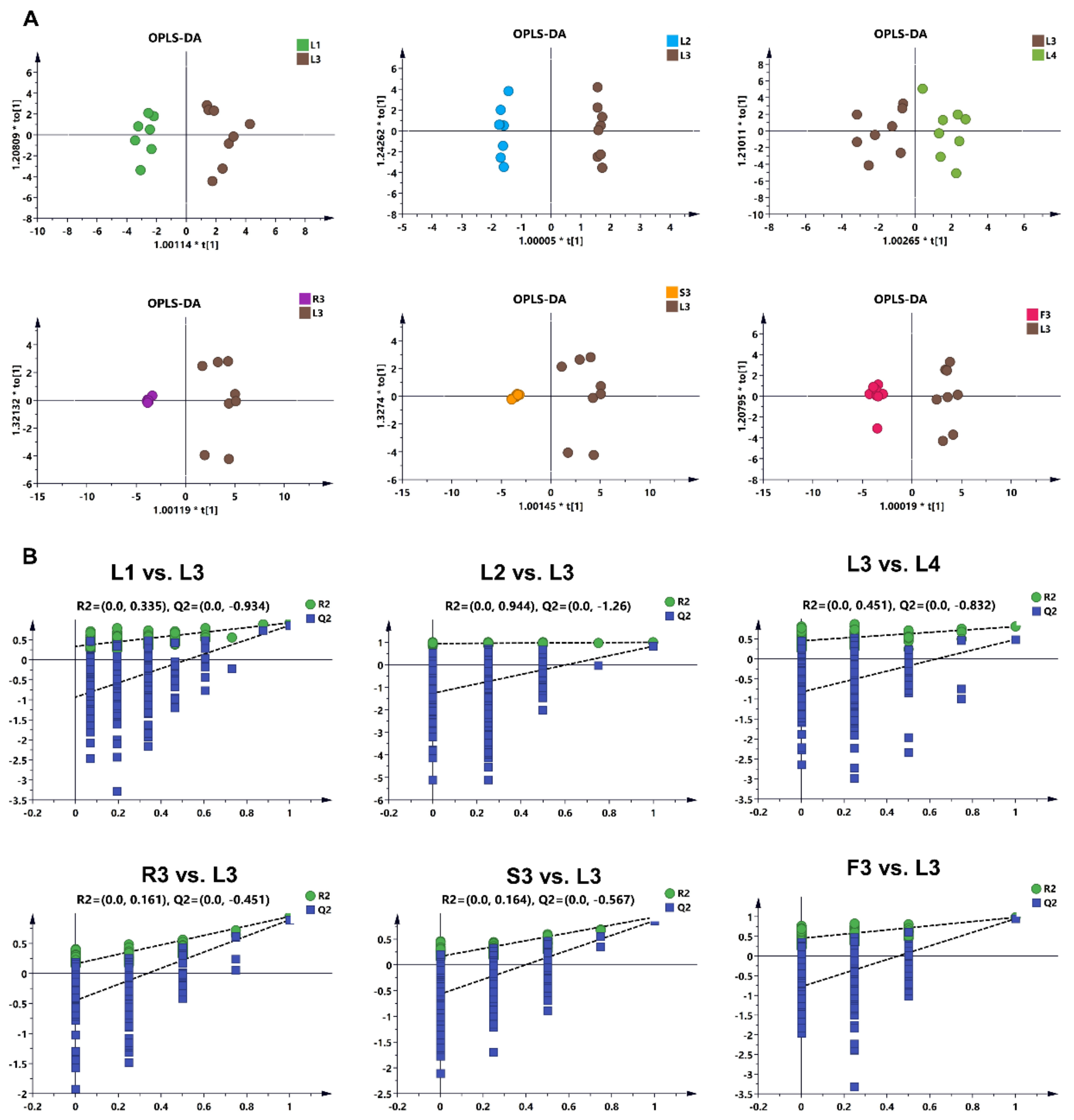

Figure S4. Score plots analysis based on UPLC-QTOF-MS data. (A) The OPLS-DA score plot of metabolite profiles L1, L2, L4, R3, S3, and F3 with L3 as the control group. Each point represents an independent biological replicate. (B) The OPLS-DA scatter plot of statistical validation obtained by 200 times permutation test, with R2 and Q2 values in the vertical axis, the correlation coefficient (between the permuted and true class) in the horizontal axis, and the ordinary least squares (OLS) line for the regression of R2 and Q2 on the correlation coefficients.

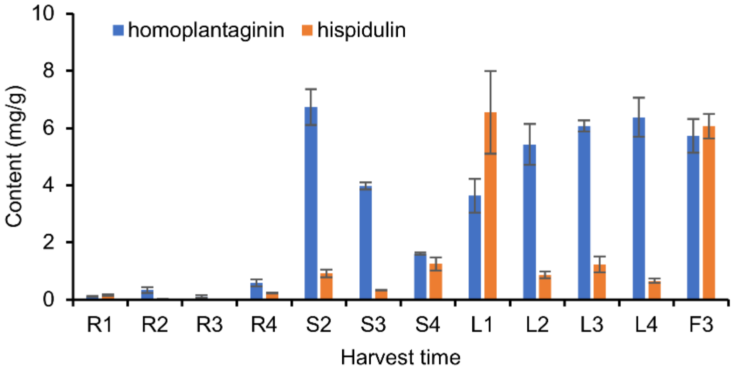

Figure S5. The content of hispidulin and homoplantaginin in *S. plebeia* with different tissues and developmental stages. Data represent Mean ± SD (n =3).

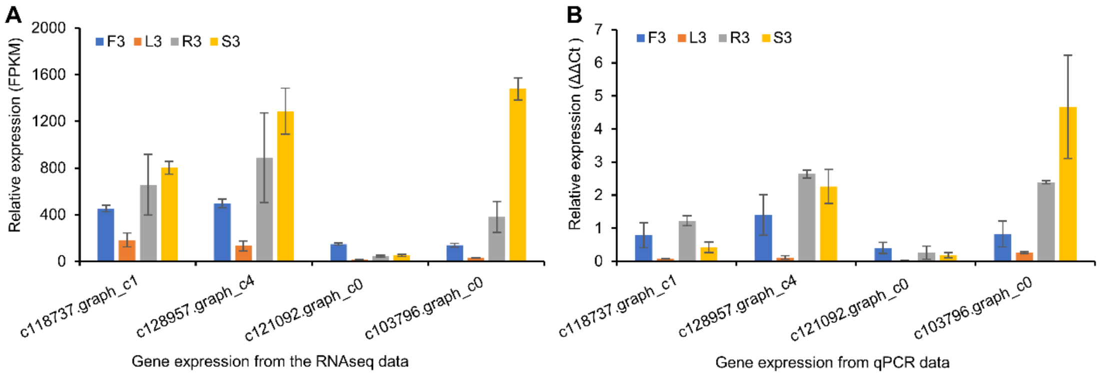

Figure S6. Validation of RNAseq by qPCR. (A) Gene expression from the RNAseq data (based on FPKM). (B) Gene expression from qPCR data (based on $2^{-\Delta\Delta Ct}$ method). Data represent Mean ± SD (n =3).

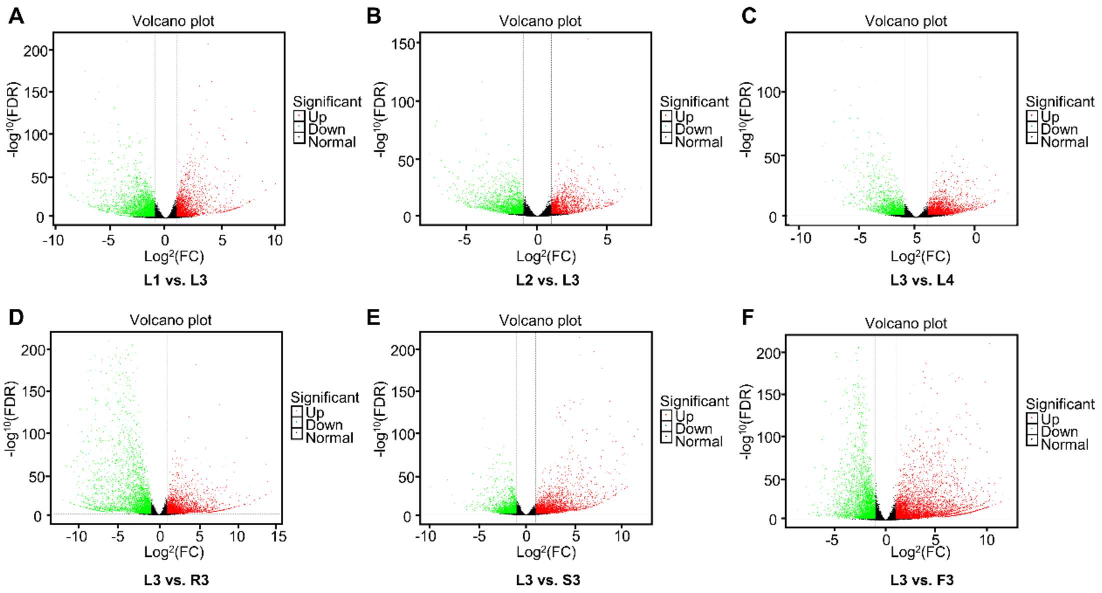

Figure S7. Volcano plots of differentially expressed genes with different tissues and developmental stages. (A) L1, leaves of basal leaf stage; L3, leaves of flower stage. (B) L2, leaves of stem elongation stage; L3 leaves of flower stage. (C) L3, leaves of flower stage; L4, leaves of ripening stage. (D) L3, leaves of flower stage; R3, root of flower stage. (E) L3, leaves of flower stage; S3, stem of flower stage. (F) L3, leaves of flower stage; F3, flower of flower stage. The groups are both in three independent biological replicates.

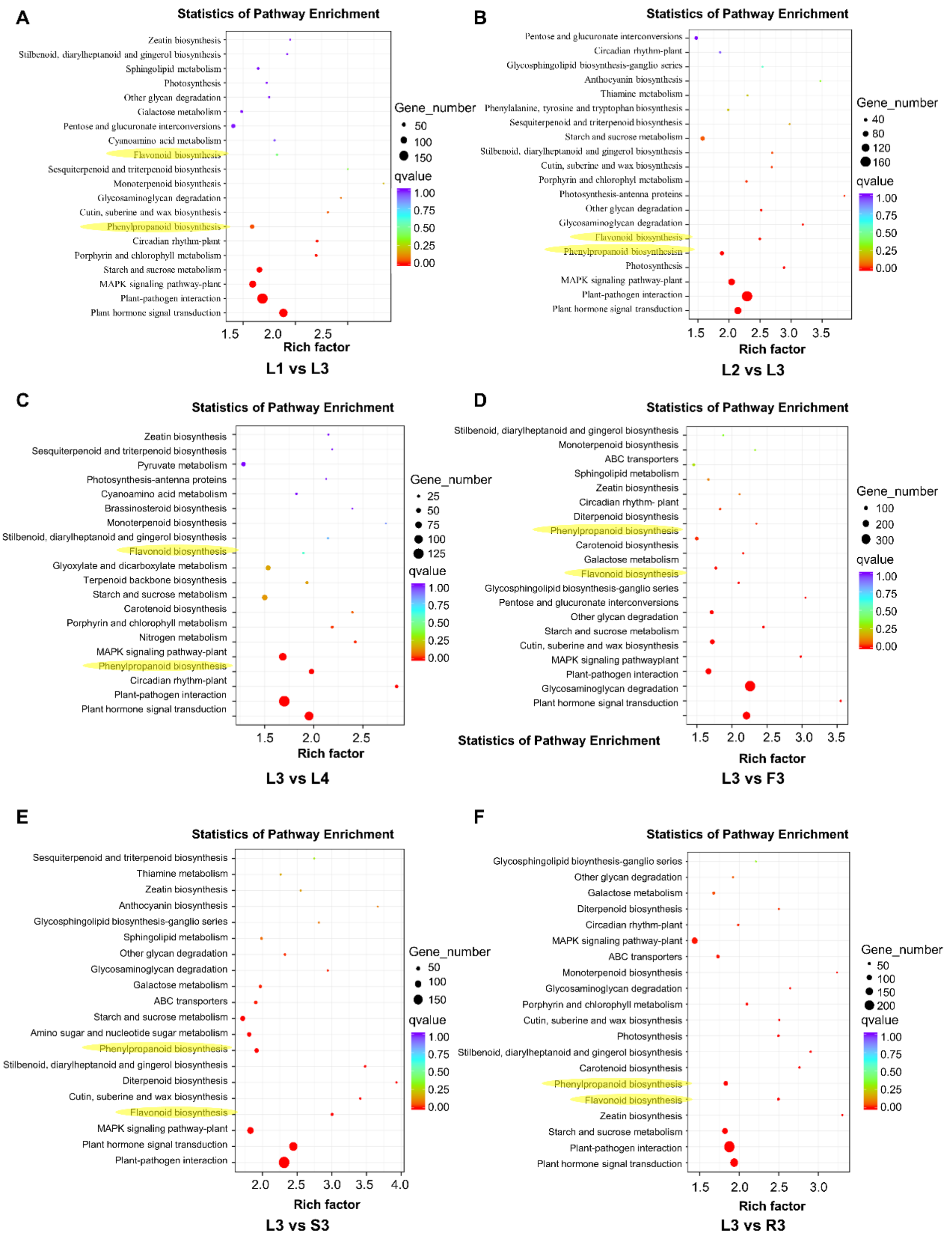

Figure S8. KEGG enrichment analysis diagram differentially expressed genes with different tissues and developmental stages. (A) L1, leaves of basal leaf stage; L3, leaves of flower stage. (B) L2, leaves of stem elongation stage; L3 leaves of flower stage. (C) L3, leaves of flower stage; L4, leaves of ripening stage. (D) L3, leaves of flower stage; F3, flower of flower stage. (E) L3, leaves of flower stage; S3, stem of flower stage. (F) L3, leaves of flower stage; R3, root of flower stage. The groups are both in three independent biological replicates.

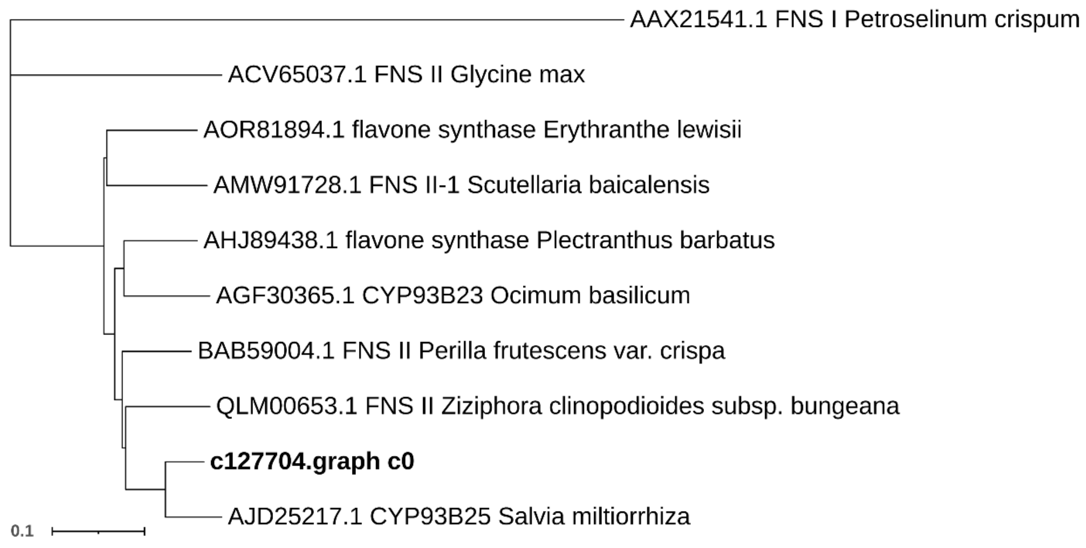

Figure S9. Phylogenetic tree of FNS. The Neighbor-Joining method was used to construct this tree with bootstrap (n=1000). AAX21541.1, Petroselinum crispum; ACV65037.1, Glycine max; AOR81894.1, Erythranthe lewisii; AMW91728.1, Scutellaria baicalensis; AHJ89438.1, Plectranthus barbatus; AGF30365.1, Ocimum basilicum; BAB59004.1, Perilla frutescens var. crispa; QLM00653.1, Ziziphora clinopodioides subsp. bungeana; AJD25217.1, Salvia miltiorrhiza.

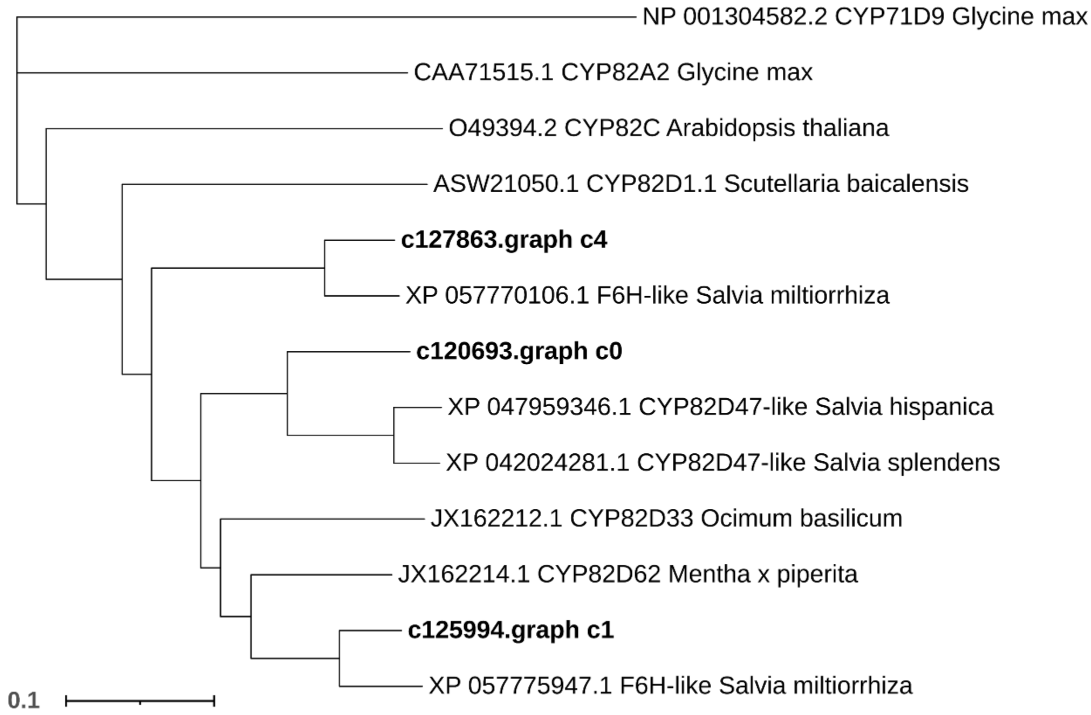

Figure S10. Phylogenetic tree of F6H. The Neighbor-Joining method was used to construct this tree with bootstrap (n=1000). NP_001304582.2, Glycine max; CAA71515.1, Glycine max; O49394.2, Arabidopsis thaliana; ASW21050.1, Scutellaria baicalensis; XP_057775947.1, Salvia miltiorrhiza; XP_047959346.1, Salvia hispanica; XP_042024281.1, Salvia splendens; JX162212.1, Ocimum basilicum; JX162214.1, Mentha × piperita; XP_057770106.1, Salvia miltiorrhiza.

Table S1. The primers used in vector construction.

ID	Primer name	Nucleotide sequence (5'-3')
c118737.graph_c1	*SpPAL* F	CTGGTGCCGCGCGGCAGCCATATGGCGGCGGAGAACGGT
	SpPAL R	AGCTTGTCGACGGAGCTCGAATTCCTAGCAGATAGGTAA
c128957.graph_c4	*SpC4H* F	TTCTCTAGACGTAAAATGGATCTCCTCCTCCT
	SpC4H R	TCCAGGCCATATGTCAAAATGATCTCGGCTTCAAAAC
c116225.graph_c0	*Sp4CL*1 F	CTGGTGCCGCGCGGCAGCCATATGGGCCAACCCGAGTCG
	*Sp4CL*1 R	AGCTTGTCGACGGAGCTCGAATTCTCAGATCAATGCAAG
c117077.graph_c0	*Sp4CL*2 F	TTATCTCCATATGACGATGTTGTCGGTGGCAG
	*Sp4CL*2 R	GGTAAGCTTTTAAGATGTGGGGGAAGTTGCT
c99751.graph_c0	*Sp4CL*3 F	GTTGGATCCATGGAGAAATCCGGATATGGC
	*Sp4CL*3 R	CCGGAATTCTTACAACTTTGATCGGACTTTGTCG
c111953.graph_c0	*Sp4CL*4 F	CTTTCTAGAGCTGGACCACACAAACAAGAAG
	*Sp4CL*4 R	CCACCTCCATATGGCAGCTCCGTTGAAATTAAAGTCT
c127981.graph_c1	*Sp4CL*5 F	CTGGTGCCGCGCGGCAGCCATATGGAGGTTCCCGCGAGG
	*Sp4CL*5 R	AGCTTGTCGACGGAGCTCGAATTCTTAGACTGCAGCTGCTAA
c126947.graph_c4	*Sp4CL*6 F	CTGGTGCCGCGCGGCAGCCATATGGCGAAATCTATCTCA
	*Sp4CL*6 R	AGCTTGTCGACGGAGCTCGAATTCTTAAAAAAATGAATA
c122523.graph_c0	*SpCHS*1 F	GCCTCTAGATTGATGTTTGCAGGTGAGAAAAC
	*SpCHS*1 R	GGTAAGCTTTTAATCTGAGTTGATGGGCACAC
c122262.graph_c0	*SpCHS*2 F	AGGAGATATACATATGATGTCGGAGGAGAT
	*SpCHS*2 R	TCCGATATCAGCCATGGTCACACCGTGAGA
c82070.graph_c0	*SpCHS*3 F	GGAGATATACATATGATGCAAAACTCCCCA
	*SpCHS*3 R	CGATATCAGCCATGGTTAACGCAGCAGGTT
c121092.graph_c0	*SpCHI* F	CTGGTGCCGCGCGGCAGCCATATGTCTGCAACGCCAACC
	SpCHI R	AGCTTGTCGACGGAGCTCGAATTCTCAGAAATCAGCGAG
c127704.graph_c0	*SpFNS* F	CGCGGATCCATGGAGCTAGTAGAAGTGGCC
	SpFNS R	CCAATTCCATATGCAAACACTAAACTACCCCGCC
c120693.graph_c0	*SpF6H1* F	CCGTCTAGACACTAGCTAGCCATGGAGTTG
	SpF6H1 R	TCTGGATCCGATGAGTCAATGCTGGAGCTG
c125994.graph_c1	*SpF6H2* F	GGTCTAGATTAGTCATGGAATTAAGCGCCG
	SpF6H2 R	CCTGAATTCCTAAGTATAGAGATTTGGTGGCAGC
c113142.graph_c1	*SpF6OMT1* F	CTGGTGCCGCGCGGCAGCCATATGGATCGGAAAACACAG
	SpF6OMT1 R	AGCTTGTCGACGGAGCTCGAATTCTCAGCTGATACGGCGGCA
c103796.graph_c0	*SpF6OMT2* F	CTGGTGCCGCGCGGCAGCCATATGGCGCAAACTGCTGCG
	SpF6OMT2 R	AGCTTGTCGACGGAGCTCGAATTCTCAGCTGATACGGCGGCA
c119249.graph_c0	*SpF6OMT3* F	CTGGTGCCGCGCGGCAGCCATATGGAAACCAAAGCAGTG
	SpF6OMT3 R	AGCTTGTCGACGGAGCTCGAATTCTTATTGGGAATTTGA
c126822.graph_c0	*SpUGT1* F	TATTTTCAGGGAGAATTCATGTTAAAAATGGAAAAC
	SpUGT1 R	ATTAACATTAGCGGCCGCTTAAGCCCGTGTAACTGC

c130898.graph_c0	*SpUGT2* F	TATTTTCAGGGAGAATTCATGGAGAAATTCGAGCTA
	SpUGT2 R	ATTAACATTAGCGGCCGCCTAGTAATCTTTATTAAA
c110923.graph_c0	*SpUGT3* F	CTGTATTTTCAGGGAGAATTCATGACAAGTCCAACTATA
	SpUGT3 R	AACTTAATTAACATTAGCGGCCGCTTAAGCGGAATTCCA
c111184.graph_c1	*SpUGT4* F	CTGTATTTTCAGGGAGAATTCATGGAAAGTATCGTACTA
	SpUGT4 R	TTAATTAACATTAGCGGCCGCTTAAGCAGAGTTCCATAT
c128814.graph_c0	*SpUGT5* F	CTGTATTTTCAGGGAGAATTCATGGATGCAAAGCAA
	SpUGT5 R	ATTAACATTAGCGGCCGCTCATTTCCTAGTACAGAT
c130729.graph_c5	*SpUGT6* F	TATTTTCAGGGAGAATTCATGGCTCCTCTTTCTTCT
	SpUGT6 R	ATTAACATTAGCGGCCGCTTATGATATAATGCTTTG
c130342.graph_c1	*SpUGT7* F	CTGTATTTTCAGGGAGAATTCATGCAAGAGCAAAGA
	SpUGT7 R	ATTAACATTAGCGGCCGCCTATTTTTTCGATATCTT
c128272.graph_c0	*SpUGT8* F	CTGTATTTTCAGGGAGAATTCATGTTTCGTGGGACG
	SpUGT8 R	ATTAACATTAGCGGCCGCTTATTTCCTACTAAGATG
c128749.graph_c0	*SpUGT9* F	CTGTATTTTCAGGGAGAATTCATGTCTGGCAATAAGTGTAAC
	SpUGT9 R	TTAATTAACATTAGCGGCCGCTCATGGCTGAGAAGCAGTTAG
c126648.graph_c0	*SpUGT10* F	TATTTTCAGGGAGAATTCATGGAGCTGGTGTTGATC
	SpUGT10 R	TTAATTAACATTAGCGGCCGCTTAGGCCATGATATCATC

Table S2. Primers for validation of gene expression by qRT-PCR.

**Primer name**	**Nucleotide sequence (5'-3')**
RT-c118737.graph_c1 F	ACCGCCACCAAGATGATCGAG
RT-c118737.graph_c1 R	AGCCCTTGAAGCCGTAGTCGAG
RT-c128957.graph_c4 F	CGGTGATCAAGGAGACACTTCGT
RT-c128957.graph_c4 R	CTCCAAGAACCTCTCGGGCCTA
RT-c121092.graph_c0 F	GACGAGAGTGACGATGATTGTGCC
RT-c121092.graph_c0 R	GCAGGTGATTGAGTGAAGAGGATG
RT-c103796.graph_c0 F	TGGATGCTGACAAAGACAACTACC
RT-c103796.graph_c0 R	ATCAGCAGCGAGAGCCTT
RT-Actin-F	TCCCTTTATGCCAGCGGTCGT
RT-Actin-R	ATCTTCATGAGGCTGTCGGT

Table S3. Plasmids and strains used in this study.

Plasmids	Description
pET32a-SpF6H1-SpUGT1	pET32a carrying *SpF6H1*, *SpUGT1* and *AtCPR*
pACYCDuet-1-SpFNS-SpF6OMT2	pACYCDuet-1 carrying *SpFNS*, *SpF6OMT2* and *AtCPR*
**Strains**	
E. coli. BL21(DE3)	
HP1	BL21(DE3) carrying *pET32a-SpF6H1-AtCPR-SpUGT1*
HP2	BL21(DE3) carrying *pACYCDuet-1-SpF6OMT2-SpFNS-AtCPR*
HP3	BL21 (DE3) carrying *pET32a-SpF6H1-AtCPR-SpUGT1* and *pACYCDuet-1-SpF6OMT2-SpFNS-AtCPR*

Table S4. Differential metabolites (DEMs) of *S. plebeia* with different tissues and developmental stages.

**L1 vs. L3**		**L2 vs. L3**		**L3 vs. L4**		**R3 vs. L3**		**S3 vs. L3**		**F3 vs. L3**	
Var ID (Primary)	VIP	Var ID (Primary)	VIP	Var ID (Primary)	VIP	Var ID (Primary)	VIP	Var ID (Primary)	VIP	Var ID (Primary)	VIP
299.1/11.0 (808)	1.95	299.1/9.8 (812)	1.15	285.0/9.5 (732)	1.36	299.1/11.0 (808)	2.06	299.1/11.0 (808)	1.93	299.1/11.0 (808)	1.23
299.1/9.8 (812)	1.62	315.1/8.1 (941)	1.67	299.1/11.0 (808)	3.15	315.1/9.7 (946)	2.10	315.1/9.7 (946)	2.21	315.1/9.7 (946)	1.49
315.1/8.1 (941)	2.02	317.1/9.0 (976)	1.85	301.1/10.5 (840)	1.49	317.1/9.0 (976)	1.34	317.1/9.0 (976)	1.43	317.1/9.0 (976)	1.26
317.1/9.0 (976)	1.28	329.2/13.9 (1049)	3.47	315.1/9.7 (946)	3.11	329.2/13.9 (1049)	1.86	329.1/11.4 (1042)	1.01	329.2/13.9 (1049)	2.56
329.2/13.9 (1049)	2.30	331.2/13.2 (1081)	1.87	317.1/9.0 (976)	1.95	331.2/13.2 (1081)	1.33	329.2/13.9 (1049)	1.90	331.2/13.2 (1081)	2.13
331.2/13.2 (1081)	1.57	343.2/13.8 (1145)	1.51	329.2/13.9 (1049)	1.54	345.2/13.2 (1174)	1.27	331.2/13.2 (1081)	1.39	343.2/13.8 (1145)	1.78
343.2/13.8 (1145)	1.14	345.2/13.2 (1174)	1.92	343.2/13.8 (1145)	1.11	359.1/6.1 (1272)	1.92	343.2/13.8 (1145)	1.05	345.2/13.2 (1174)	2.38
345.2/13.2 (1174)	1.53	359.1/6.1 (1272)	1.52	359.1/6.1 (1272)	2.02	359.2/13.9 (1283)	1.54	345.2/13.2 (1174)	1.33	349.2/13.4 (1223)	1.43
359.1/6.1 (1272)	3.17	359.2/13.9 (1283)	2.85	359.2/13.9 (1283)	1.23	447.1/3.6 (1805)	1.83	359.1/6.1 (1272)	2.05	359.1/6.1 (1272)	1.43

359.2/13.9 (1283)	1.84	447.1/3.6 (1805)	1.90	447.1/3.6 (1805)	1.52	461.1/6.6 (1907)	3.04	359.2/13.9 (1283)	1.61	359.2/13.9 (1283)	1.93
447.1/3.6 (1805)	1.18	449.1/3.3 (1830)	1.17	461.1/6.6 (1907)	1.79	463.1/2.5 (1923)	1.74	447.1/3.6 (1805)	1.92	431.1/5.5 (1731)	1.31
461.1/6.6 (1907)	1.64	461.1/6.6 (1907)	1.13	463.1/2.5 (1923)	1.40	463.1/5.9 (1928)	1.24	461.1/6.6 (1907)	2.40	447.1/3.6 (1805)	1.78
463.1/2.5 (1923)	1.51	463.1/2.5 (1923)	1.89	463.1/5.9 (1928)	1.40	477.1/4.4 (2013)	2.72	463.1/2.5 (1923)	1.83	461.1/6.6 (1907)	1.87
463.1/5.9 (1928)	1.15	479.1/3.9 (2046)	1.55	477.1/4.4 (2013)	1.79	479.1/3.9 (2046)	1.80	463.1/5.9 (1928)	1.27	463.1/2.5 (1923)	1.79
477.1/4.4 (2013)	1.45			479.1/3.4 (2047)	1.56			477.1/4.4 (2013)	2.76	463.1/5.9 (1928)	1.20
479.1/3.9 (2046)	2.22			479.1/3.9 (2046)	2.34			479.1/3.9 (2046)	1.89	477.1/4.4 (2013)	2.40
										479.1/3.9 (2046)	1.91

Table S5. Overall quality assessment of raw data from RNA-Seq.

SampleID	ReadSum	BaseSum	GC(%)	N(%)	Q20(%)	Q30(%)
F3-1	25771415	7.69E+09	49.4	0	98.35	95.11
F3-2	24512991	7.32E+09	48.63	0	98.17	94.66
F3-3	24514459	7.32E+09	48.47	0	98.14	94.57
L1-1	24663619	7.35E+09	49.69	0	98.36	95.14
L1-2	24366831	7.27E+09	49.62	0	98.28	94.9
L1-3	24817806	7.41E+09	49.4	0	98.18	94.71
L2-1	23948797	7.16E+09	49.96	0	98.1	94.46
L2-2	25204431	7.54E+09	49.27	0	98.11	94.51
L2-3	26011583	7.78E+09	49.42	0	98.22	94.78
L3-1	26348631	7.89E+09	48.56	0	98.28	94.92
L3-2	25344880	7.57E+09	48.57	0	98.32	95.03
L3-3	26194166	7.84E+09	48.65	0	98.37	95.11
L4-2	27554620	8.23E+09	48.43	0	98.38	95.15
L4-3	27732789	8.30E+09	48.78	0	98.13	94.55
L4-4	26122541	7.81E+09	48.4	0	98.34	95.04

R3-1	25378612	7.59E+09	47.88	0	98.23	94.78
R3-2	26550975	7.95E+09	47.9	0	98.18	94.65
R3-3	28618091	8.56E+09	47.3	0	98.33	95.03
S3-1	25694348	7.69E+09	48.7	0	98.17	94.67
S3-2	24309491	7.27E+09	48.82	0	98.2	94.67
S3-3	26462613	7.90E+09	48.87	0	98.13	94.6

Table S6. Statistics of *S. plebeia* splicing transcriptome.

Term	Number	Mean Length	Total Length	N50
ranscript	140509	1583.39	222480119	1503
Unigene	58905	1161.28	68405575	1754

Table S7. Functional annotation of the *S. plebeia* transcriptome.

Database	Number
COG	13330
GO	33956
KEGG	25388
KOG	25279
Pfam	33267
Swissprot	22987
TrEMBL	38304
eggNOG	29485
NR	41341
All	44185

Table S8. Differentially in the expressed genes.

DEG_Set	All_DEG	up-regulated	down-regulated
L1-1_L1-2_L1-3_vs_L3-1_L3-2_L3-3	5112	2201	2911
L2-1_L2-2_L2-3_vs_ L3-1_L3-2_L3-3	3798	1644	2154
L3-1_L3-2_L3-3_vs_ F3-1_F3-2_F3-3	7896	4665	3231
L3-1_L3-2_L3-3_vs_ L4-2_L4-3_L4-4	4211	2370	1841
L3-1_L3-2_L3-3_vs_R3-1_R3-2_R3-3	6366	2923	3443
L3-1_L3-2_L3-3_vs_ S3-1_S3-2_S3-3	4471	2681	1790

Table S9. KEGG analysis of structural genes in the flavonoid biosynthesis pathway.

Gene ID	Gene name	KEGG	FPKM values																				
			L1-1	L1-2	L1-3	L2-1	L2-2	L2-3	L3-1	L3-2	L3-3	L4-1	L4-2	L4-3	S3-1	S3-2	S3-3	R3-1	R3-2	R3-3	F3-1	F3-2	F3-3
c128957.graph_c	C4H	ko0094	85.69	101.9	127.7	657.2	287.5	391.7	92.08	193.6	113.7	226.6	92.15	74.37	1433.5	1419.6	1010.2	970.6	1312.9	385.0	447.9	533.11	510.5
c103796.graph_c	CCOMT	ko0094	52.08	45.2	86.57	103.3	61.78	33.86	28.26	27.2	31.94	415.2	170.2	73.69	1342.8	1532.5	1556.7	551.9	362.6	229.6	140.3	159.99	118.6
c113142.graph_c	CCOMT	ko0094	14.11	8	8.85	3.83	5.76	11.65	2.87	1.83	6.1	0.58	1.91	0.8	51.58	78.33	419.05	26.53	10.95	5.74	13.97	15.65	52.04
c119249.graph_c	CCOMT	ko0094	1.26	1.24	0.77	1.25	1.32	0.85	1.58	1.16	1.23	2.65	1.81	1.7	2.36	3.01	1.33	3.25	3.58	4.43	6.06	6.63	5.72
c107876.graph_c	OMT	ko0094	0.52	0.49	0.41	1.24	1.25	0.8	0.55	0.67	0.49	0.34	2.22	0.12	36.23	30.86	26.22	104.8	100.96	45.04	2.44	2.07	3.86
c118570.graph_c	OMT	ko0094	43.31	44.72	55.46	380.0	135.3	205.8	30.44	50.47	36	6.69	8.8	4.91	326.19	330.51	292.77	83.7	121.07	37.05	247.1	284.77	253.8
c124024.graph_c	CHI	ko0094	110.6	141.2	126.6	512.1	199.4	149.5	73.62	86.09	40.78	41.99	19.74	23.05	86.48	124.46	60.88	18.79	51.06	6.33	287.0	347.49	182.4
c122523.graph_c	CHS	ko0094	6.43	3.96	6.6	17.39	15.39	12.9	14.85	22.56	27.21	1.82	1.86	2.77	5.99	6.54	7.16	0	0.15	0.42	17.11	18.44	24.06
c122262.graph_c	CHS	ko0094	562.3	420.6	214.5	800.7	720.0	387.0	18.6	35.72	52.15	1.6	2.02	0.2	83.85	286.17	428.35	22.33	0.94	3.91	768.2	1073.6	976.4
c82070.graph_c0	CHS	ko0094	0	0	0	0.21	0.05	0.05	0	0.04	0	0	0.05	0	0.15	0.11	0	1.62	1.44	1.88	47.83	17.15	25.95
c119231.graph_c	DFR	ko0094	9.79	10.62	11.76	5.63	1.74	2.6	0.39	0.45	0.71	4.21	0.53	0.24	0.43	0.82	0.28	0.19	0.08	0.21	16.18	12.87	11.33
c83812.graph_c0	DFR	ko0094	0.79	0.62	0.46	7.7	5.79	12.36	1.45	1.13	1.58	0.23	1.12	0.1	7.05	8.78	2.97	0.34	0.36	0.34	162.7	188.75	209.4
c108967.graph_c	F3'5'H	ko0094	0.03	0	0.03	0	0	0	0	0	0	0	0	0	0	0	0	0.03	0	0	65.53	106.85	117.9
c115905.graph_c	F3H	ko0094	13.48	18.99	18.76	6.7	9.57	8.47	8.58	8.37	6.28	8.81	5.24	5.33	3.55	3.47	1.74	3.03	4.31	1.59	17.67	21.75	27
c130349.graph_c	F3H	ko0094	0.36	0.13	0.2	1.59	1.56	3.31	0.76	0.27	1.42	0.56	0.67	0	3.85	5.79	1.94	0.12	0.33	0.55	120.4	132.54	139.2
c100929.graph_c	F3H	ko0094	1.82	0.87	1.29	1.32	1.04	0.67	0.52	1.11	0.39	0.05	0	0	0.39	0.33	0	0	0	0	3.99	7.3	6.19
c122302.graph_c	FLS	ko0094	8.43	12.37	11.97	12.77	20.87	24.5	30.93	34.06	38.21	7.22	16.08	7.58	22.3	22.85	16.79	4.84	4.44	5.03	6.79	9.06	5.18
c110923.graph_c	UGT	ko0094	39.89	41.02	24.48	12.81	19.98	22.19	15.29	15.95	32.84	5.22	6.7	15.64	15.52	11.09	10.66	22.29	11.41	17.41	20.82	23.66	26.36
c101658.graph_c	UGT	ko0094	0.05	0.05	0.05	0.11	0.16	0	0.29	0	0	0.19	0	0.05	0.1	0	0.1	0.15	0.19	0.05	5.88	6.22	5.72
c110923.graph_c	UGT	ko0094	77.27	77.12	68.9	43.38	45.64	45.29	46.41	33.84	67.13	12.61	19.72	29.1	24.02	25.44	24.92	30.16	25.12	35.58	37.09	38.2	40.12
c111184.graph_c	UGT	ko0094	3.3	2.51	2.29	7.28	4.05	3.43	2.37	1.98	2.31	1.76	0.52	1.42	43.4	61.23	56.72	6.82	7.71	7.06	11.05	11.21	10.45
c115214.graph_c	UGT	ko0094	2.47	2.43	1.81	6.28	4.51	3.98	3.66	3.73	3.78	24.92	6.51	8.45	7.69	10.05	4.96	15.65	12.6	14.2	11.9	17.81	14.75
c115878.graph_c	UGT	ko0094	141.7	126.0	114.1	65.76	123.6	103.5	138.2	102.4	136.6	86.83	169.1	190.3	82.23	87.89	66.99	0.74	0.05	0.54	23.53	36.56	27.98

c118512.graph_c	UGT	ko0094	4.54	4.46	4.74	7.55	3.53	9.62	3.88	5.03	7.63	6.84	12.67	5.15	5.33	5.05	3.26	54.49	41.86	34.32	17.71	22.32	18.33
c118635.graph_c	UGT	ko0094	0.08	0.09	0.29	0	0.08	0.04	0.12	0.18	0.47	0.53	1.31	1.35	0.3	0.31	0.19	0.06	0.06	0.15	0.38	0.38	0.34
c127652.graph_c	UGT	ko0094	0.37	0.2	0	0.38	0.18	0.38	0.23	0.24	2.06	0.29	2.09	0.22	0.05	0.37	0	7.46	6.28	6.45	2.46	2.6	4.93
c128814.graph_c	UGT	ko0094	14.24	25.94	31.56	9.33	6.83	7.05	19.5	17.2	12.09	60.83	28.4	28.99	11.29	9.24	7.05	41.65	36.7	61.29	11.45	13.12	10.93

Table S10. Unigenes were selected as candidates gene.

Gene ID	Gene name
c128957.graph_c4	C4H
c129866.graph_c0	C4H
c103796.graph_c0	CCOMT
c113142.graph_c1	CCOMT
c119249.graph_c0	CCOMT
c107876.graph_c0	OMT
c118570.graph_c0	OMT
c124024.graph_c0	CHI
c121092.graph_c0	CHI
c122523.graph_c0	CHS
c122262.graph_c0	CHS
c82070.graph_c0	CHS
c110923.graph_c0	UGT
c101658.graph_c1	UGT
c110923.graph_c1	UGT
c111184.graph_c1	UGT
c115214.graph_c0	UGT
c115878.graph_c0	UGT
c118512.graph_c0	UGT
c118635.graph_c0	UGT
c127652.graph_c0	UGT
c128814.graph_c0	UGT
c130898.graph_c0	UGT
c126822.graph_c0	UGT
c127870.graph_c1	UGT
c130729.graph_c5	UGT
c130342.graph_c1	UGT
c128272.graph_c0	UGT
c108895.graph_c0	UGT
c126648.graph_c0	UGT
c118737.graph_c1	PAL
c113652.graph_c0	PAL
c106045.graph_c0	4CL
c108148.graph_c1	4CL
c111953.graph_c0	4CL
c115053.graph_c0	4CL
c117077.graph_c0	4CL
c117269.graph_c0	4CL
c119638.graph_c0	4CL
c119899.graph_c0	4CL
c122490.graph_c0	4CL
c124442.graph_c1	4CL
c126265.graph_c4	4CL
c126467.graph_c1	4CL
c126776.graph_c1	4CL
c126947.graph_c4	4CL

c127981.graph_c1	4CL
c128784.graph_c0	4CL
c128850.graph_c0	4CL
c127704.graph_c0	FNS
c120693.graph_c0	F6H
c125994.graph_c1	F6H
c127863.graph_c4	F6H
